# Characterization of Biobased Polymers at the Gas–Solid Interface—Analysis of Surface and Bulk Properties during Artificial Degradation

**DOI:** 10.1021/acs.est.4c10925

**Published:** 2025-04-19

**Authors:** T. Borgmeyer, Y. Kupper, M. J. Rossi, J. S. Luterbacher, C. Ludwig

**Affiliations:** † École Polytechnique Fédérale de Lausanne (EPFL), GR-LUD, School of Architecture, Civil and Environmental Engineering (ENAC IIE), Station 6, CH-1015 Lausanne, Switzerland; ‡ École Polytechnique Fédérale de Lausanne (EPFL), Laboratory of Sustainable and Catalytic Processing (LPDC), Institute of Chemical Sciences and Engineering (ISIC), Station 6, CH-1015 Lausanne, Switzerland; § Paul Scherrer Institute (PSI), Center for Energy and Environmental Sciences, CH-5232 Villigen PSI, Switzerland

**Keywords:** bioplastics, surface functionality, polymer degradation, surface and bulk material properties, gas-phase titration

## Abstract

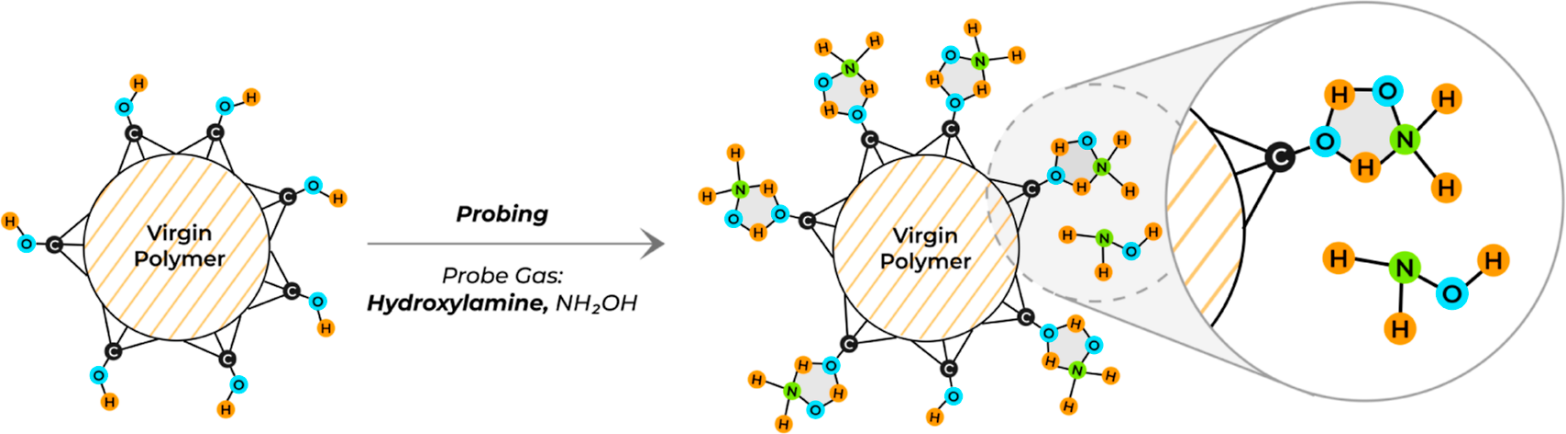

Accurately assessing the interfacial composition and reactivity of (bio)polymers under controlled but environmentally relevant conditions remains challenging. This study explores the evolution of surface functionalities of novel polyesters Poly(Butylene Xylose) (PBX) and Poly(Alkyl Xylose Amide) (PAXA) under artificial degradation conditions. Employing a Knudsen Flow Reactor (KFR) and a gas-titration approach, we systematically analyze the chemical transformations occurring at the polymer’s solid–gas interface. Both polymers are derived from the same functionalized lignocellulosic sugar building block, Diglyoxylic Acid Xylose (DGAX). Virgin (V), long-term UV (UV), and short-term Ozone (O_3_) exposures induce specific but differing alterations in molecular integrity and surface reactivity, resulting in notable shifts in material properties and polymer structure. Contrary to the assumption that degradation increases specific surface area (SSA) and reactivity, our results reveal cases where the SSA decreases, with reactivity either increasing or decreasing based on the reactive groups available at the interface. Both polymers exhibited increased water affinity, acidification, and ozone reactivity following UV exposure. Interfacial reactivity, assessed with trifluoroacetic acid, hydroxylamine, and nitrogen dioxide, increased for PBX_UV_ but decreased for PAXA_UV_. Surface hydroxyl groups in PAXA reduced 5-fold under short-term ozone and long-term UV exposure, while bulk transport kinetics of hydroxylamine altered with long-term degradation, though ozone exposure left transport mechanisms unaffected. This combined interfacial and bulk analysis approach advances our understanding of (bio)plastic degradation. It examines the role of degraded polymers as potentially more or less reactive vectors for chemicals and organisms upon environmental release while also demonstrating that polymer degradation initiates significantly earlier than previously assumed.

## Abstract 2

Polymer particle degradation and induced surface functionality changes are crucial for predicting environmental impact, particularly their role as reactive vectors and pollutant carriers.

## 1 Introduction

A staggering 400.3Mt of plastics were produced worldwide in 2022, with production steadily increasing.^1^ Approximately 0.5% of this amount is considered to be Bioplastic materials.^2^ Researchers estimate around 10Mt of plastic materials being lost and littered into the environment in 2015 alone, causing several studied problems,^3^ since polymers are subject to various (a)biotic degradation mechanisms, when (un)intentionally ending up in the environment at the end of their life cycle.^4^

Plastic fragments commonly referred to as macro- or microplastics vary in surface to volume ratio and reactivity when exposed to environmental conditions.^3^ Techniques to detect and analyze their unknown composition, assess their degradation state, and predict their reactivity are frequently not only described in the literature^5,6^ but also show distinct limitations.^7^ Commonly utilized methods are limited to the analysis of bulk material properties, due to limitations in sensitivity and excessive physical penetration depth or in some cases in the destructive nature of the chosen method of analysis.^8^ However, the surface, or “interface”, is the location of the initial interaction and is the gateway for potential environmental degradation of plastics.^7^ W. Chen created controlled hydrolysis conditions but were not able to investigate small changes using Attenuated Total Reflectance-Fourier Transform Infrared (ATR-FTIR).^9^ Additional surface-sensitive methods for the investigation of the interface of polymers have previously been applied by Z. Chen using sum frequency generation vibrational spectroscopy on a variety of polymers but has proven to be of little general use.^10^

The Knudsen Flow Reactor (KFR) enables testing for surface functional groups (SFGs) of inorganic and organic samples using a selection of probe gases.^7,11−13^ A heterogeneous chemical titration approach is used to test for acidic, basic, reducing, and hydroxyl surface functionalities at the polymer solid/gas interface.^14^ Consequently, the surface may be characterized and changes in surface composition induced by selected long- and short-term degradation processes monitored and quantified.^7^ In addition, the surface coverage of SFGs and the probe gas uptake kinetics may be studied.^15^

This study shows for the first time the differences in interfacial reactivity of two unmodified biobased polymers upon pre/post laboratory UV and ozone degradation and the impact that these two abiotic degradation factors have on the bulk and surface properties of the biopolymers.^4^ Sample materials were polymerized using different synthesis approaches and showed different material properties while sharing the common monomeric building block of the copolymer DGAX.^16,17^ Results of commonly utilized methods ATR-FTIR, Nuclear Magnetic Resonance (NMR), and Size Exclusion Chromatography-Multiple Angle Light Scattering (SEC-MALS) have been applied and combined with the highly sensitive gas-titration approach of the KFR. The results indicate that long-term laboratory UV degradation significantly impacts material properties of both polymers on different time scales while also significantly changing the interfacial reactivity. Conversely, short-term O_3_ exposure led to no significant changes in material properties but changed the surface functionality in a similar fashion that UV degradation does, when tested for i-OH using hydroxylamine (HA) as a probe gas, as explained by Borgmeyer.^7,18^

In this work, “degradation” refers to all applicable forms of aging, weathering, structural disintegration, and other processes affecting polymers. More broadly, it encompasses any conditions that might impact the polymeric material’s intrinsic properties (such as changes in structure and chemical composition).

## 2 Methods

**Figure 1:**
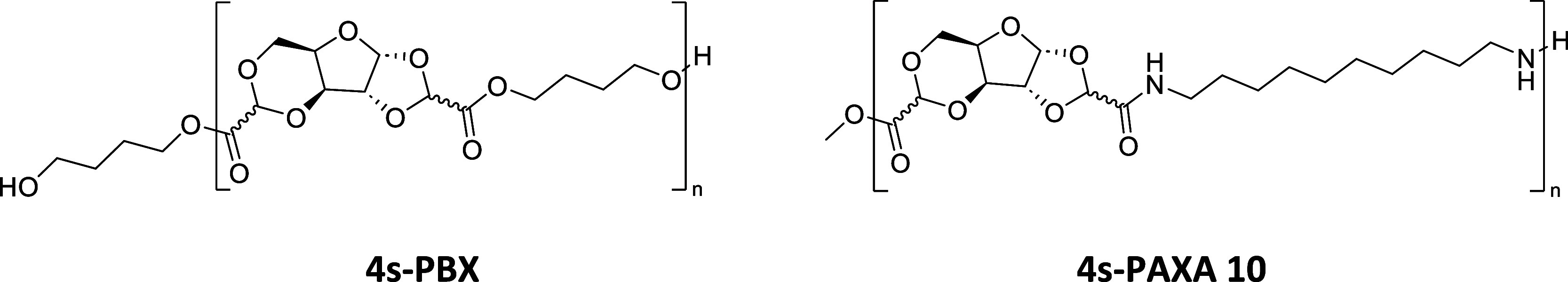
Structure of 4s-PBX (left) and 4s-PAXA 10 (right).

The polymers investigated in this study were developed and synthesized in the Laboratory of Sustainable and Catalytic Processing (LPDC), EPFL. Undegraded PBX (PBX_V_), a polyester, and PAXA (PAXA_V_), a polyamide, are displayed in Figure 1. Both (bio)polymers are based on the identical monomeric backbone structure DGAX, extracted from nonedible wood biomass, and details on the process and synthesis may be found in Manker 2022 and 2024.^16,17^

A benchtop “CryoMill” by Retsch was used for the polymer particle preparation. The SSA, based on the Brunauer–Emmett–Teller (BET) theory, was measured using a Micromeritics 3Flex instrument and nitrogen gas. Scanning electron microscopy (SEM) images were taken for the visual aspect of the particles using a ZeissGeminiSEM 300 for imaging. A Beckman Coulter LS 13 320 Laser Diffractometer was used to perform Particle Size Analysis (PSA). For artificial UV degradation, a solar simulator (Atlas Ametek, Inc., Model Suntest XLS+, Lamp NXE 1700, equipped with the Sun Cool cooling system) was utilized. Parameters were set to 765 W/m^2^ without an UV filter installed at a wavelength range of 250–800 nm (Figure S1). The UV degradation of the polymers was performed by evenly spreading powders on Pyrex Ø 9 cm (63.62 cm^2^) Petri dishes, covered with Ø 10 cm, 0.2 cm thickness fused silica wafers (Siegert Wafer, GL24010). Silica wafers protect the powders from being carried away by the cooling air flow across the samples to keep a constant temperature inside the solar simulator and simultaneously transmit all UV light. The UV chamber setup is displayed in Figures S2 and S3. The UV degradation equals 307 days under nontropical conditions at 10 W/m^2^ for PBX_UV_ and 5.4 years for PAXA_UV_, calculated according to Delre.^19^ The ATR-FTIR polymeric fingerprints were analyzed using a PerkinElmer Spectrum 2, configured with the “UATR Two” accessory. The testing and analysis principle for the interfacial reactivity, alongside KFR equipment modifications were thoroughly explained by Iannarelli.^12^ Adsorption and desorption experiments were performed using several probe gases to characterize and map the SFGs present: Water vapor (H_2_O) was used to test the water sorption affinity/hydrophilicity of the polymer, Hydroxylamine (HA, (NH_2_OH)) (to test) for surface OH (i-OH) groups, Trifluoroacetic acid (TFA, (CF_3_CO_2_H)) for basic sites, Trimethylamine (TMA, (N(CH_3_)_3_)) for acidic sites, Ozone (O_3_) for all, and Nitrogen Dioxide (NO_2_) for weakly reducing sites of the molecular interface. Probe gases were synthesized as described by Setyan,^13^ purchased in highest purity and checked for quality beforehand using the KFR-Mass Spectrometer (KFR-MS) setup. O_3_-aged polymer samples for the short-term aging experiments using the KFR technique were spread out in identical glass Petri dishes, and a flow of ≈6.35 × 10^15^ # molecule/s of O_3_ was set across the sample for 20 min (Total Dose: ≈7.62 × 10^18^ # molecules of O_3_), which represents 36.15 days at an assumed average O_3_ concentration of 100 ppb (eq S1). Ultraviolet–Visible-Near Infrared Spectroscopy (UV–vis–NIR) was used to measure the optical change of the polymers by using a Shimadzu UV-3600 spectrophotometer in reflection–absorption mode. Samples for Size Exclusion Chromatography–Multi-Angle Laser Scattering (SEC-MALS) were left overnight in hexafluoro isopropanol (HFIP, Sigma-Aldrich) for depolymerization and hence solubilization and filtered using a 0.45 μm PTFE syringe filter. Proton-(^1^H−) and Diffusion-Ordered-(2D-DOSY-) NMR analysis was performed using a Bruker Avance III 400 MHz spectrometer with a BBFO-plus probe. Samples were dissolved in *d*_6_-dimethylsulfoxide (DMSO, Sigma-Aldrich), left overnight, and analyzed without filtration.

Technical Details for all methods and protocols used, especially on the KFR, may be found in the SI or additional literature.^12−14,18,20^ In the context of KFR and degradation-induced changes in surface functionality, the term “reactivity” is used to explain the total amount of probe gas molecules lost upon sample exposure, a reaction rate (# molecules s^–1^). Consequently, the uptake of molecules of approximately 10^12^ may be considered low.

## 3 Results

Preliminary experiments have shown sintering followed by melting of PBX at approximately 100 h of laboratory UV degradation; therefore, the UV degradation PBX_UV_ was halted after 96 h, and all sample values are displayed in Table 1.

**Table 1:** Change of BET-Values Pre/Post UV Degradation: PBX_UV_ Accumulated a Radiant Exposure of 265.4 MJ/m^2^ after 96 h, While PAXA_UV_ Experienced 618 h of UV Degradation Accumulating a Total of 1701.5 MJ/m^2^^a^

Sample	BET-Value (m^2^/g)	Total Radiant Exposure (MJ/m^2^)	Remaining SSA (%)
PBX_V_	1.15		100
PBX_UV_	0.3	265.4	26.4
PAXA_V_	0.57		100
PAXA_UV_	0.23	1701.5	39.6

a After artificial UV degradation, BET-values of both polymers decreased significantly: PBX_UV_ shows a decrease to 26.4%, while PAXA_UV_ decreases to 39.6% of its initial BET-value, displayed in the column “Remaining SSA”.

**Figure 2:**
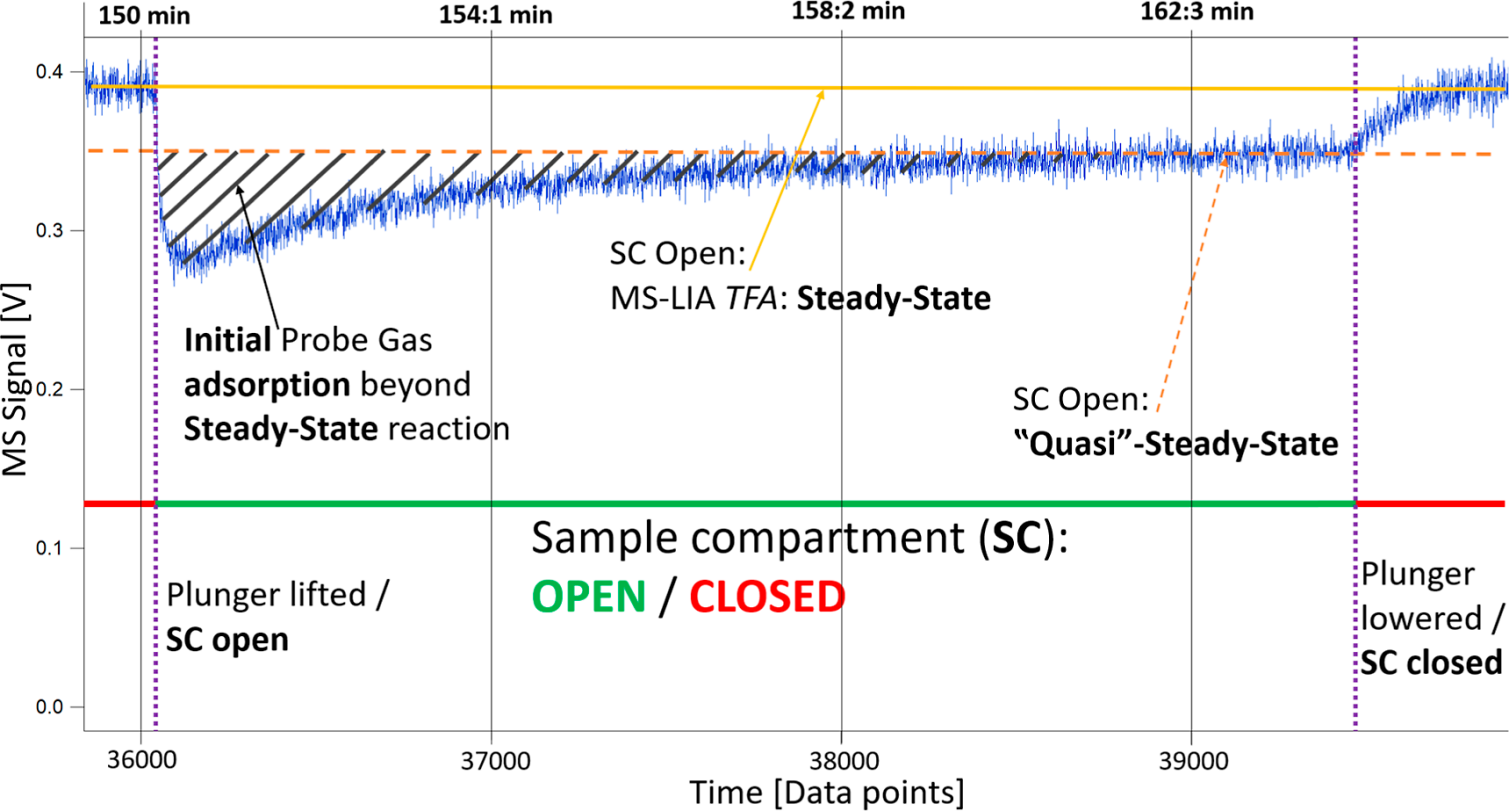
Overview of an uptake reaction of PBX_UV+O3_ when exposed to a HA flow rate of 5.5 × 10^15^ # molecule/s, at the 1 mm escape orifice: The solid yellow line indicates the initial flow of HA molecules, while the voltage (*y*-axis) is directly proportional to the flow rate of molecules detected by the MS.

The BET-values of PBX_V_ and PAXA_V_ differ by approximately a factor of 2 but can still be considered small compared to, for example, diesel soot, which has a value of 333.18 m^2^/g.^11^ The surface reactivities have been assessed according to the published KFR chemical kinetics formalism and led to the determination of the initial uptake of probe gas molecules. Polymer-steady-state-specific heterogeneous rate coefficient of uptake (*k*_het-StSt_ in s^–1^) and uptake probability per SSA (γ_BET-StSt_), as explained by Mirghaffari, have been calculated.^15^ Upon opening of the SC, the sample is exposed to a quantified constant flow of probe gas molecules. These molecules either react with gas-specific SFGs or circulate unreacted within the KFR until detected by the MS. The total flow consists of molecules reaching the MS (*k*_esc_) and molecules reacting with the sample (*k*_het_) within a chosen gas residence time, while both reactions compete. The reactions of probe gas molecules with the SFGs of the sample material are either based on adsorption (*k*_a_) and eventual desorption (*k*_d_) mechanisms—all physicochemical terms used throughout this work are displayed and explained in further detail in Figure S4. A typical gas molecule uptake experiment is shown and explained in Figure 2, using PBX_UV__+O3_ as the sample material and HA as the probe gas. A detailed KFR setup description and workflow when working with plastic particles has been presented elsewhere.^7,12^ Reactions of TFA with PAXA_V_ and PAXA_UV_ and consequently differences in reactivity are presented in Figures S7 and S8.. A detailed KFR setup description and workflow when working with plastic particles has been presented elsewhere.^7,12^ React if TFA with PAXA_V_ and PAXA_UV_ and consequently differences in reactivity are presented in Figures S7 and S8.

Figure 2 presents at time point 36.04 × 10^3^ ≈ 150 min (4 Hz acquisition frequency) the manual opening of the SC (purple dashed line) by lifting the plunger, and the consequent voltage drop results from the initial reactivity of HA with PBX_UV+O3_. Once fast reacting SFGs are saturated, the number of molecules is detected by the MS and therefore the voltage signal steadily increases until the initial flow is reached again or a new “quasi”-steady-state (orange dashed line) has emerged, which suggests a relatively slow transport mechanism into the bulk of the material (*k*_het-StSt_). The black hashed area illustrates the yield of HA molecules adsorbed by SFGs in contrast to those incorporated via mass bulk transfer. The dashed orange dividing line therefore separates the yield of HA taken up by the interface in the initial process from the slower uptake via bulk mass transfer across the interface occurring on a longer time scale. After approximately 38.5 × 10^3^ ≈ 160 min, SFGs reach saturation, and any additional loss of molecules is exclusively due to bulk transport. At the time point 39.46 × 10^3^ ≈ 164 min, the plunger was lowered (purple dashed line) and the SC closed. Consequently, the previous steady-state flow of molecules was re-established (initial HA level). A schematic drawing of the KFR is shown in Figure S4, and a complete adsorption and desorption experiment is shown and explained in Figures S5 and S6.^18^

Two kinds of experiments were carried out using the KFR: at first, a chemical mapping of SFGs present on the virgin and UV-degraded polymer surface was performed using H_2_O, HA, NO_2_, TFA, TMA, and O_3_ as probe gases. In a second step, the short-term sensitivity of the method was tested by making use of the environmentally relevant gas O_3_ as a proxy oxidative degradation reagent. Following O_3_ degradation, HA was used to quantify the change of i-OH group abundance. HA was selected because it demonstrated the highest uptake following the initial SFG mapping, as previously noted by Borgmeyer.^7^ Changes in the total number of molecules taken up were quantified for both polymers in both degradation states (“_UV_” and “_O3_”). Table 2 shows the H_2_O uptake of all four samples; it serves as an example for all subsequent KFR tables presented throughout the manuscript and in Table S1.

**Table 2:** H_2_O Uptake of PBX_V_, PBX_UV_, PAXA_V_, and PAXA_UV_: Normalized Total Uptake, the Ratio between Virgin and UV-Degraded Samples, Inter-Degradation-State Factor, Intra-Polymer-Factor, and Presence of “Quasi”-Steady-State Including *k*_het-StSt_ and γ_BET-StSt_

**Probe Gas:** H_2_O	**PBX_V_**	**PBX_UV_**		**PAXA_V_**	**PAXA_UV_**
Uptake (# of molecule/cm^2^)	6.28 × 10^12^	4.87 × 10^13^		1.60 × 10^13^	9.48 × 10^13^
Increase/Decrease w/r virgin polymer	1	7.75		1	5.93
Factor between virgin polymers	2.55				
Factor between degraded polymers		1.95			
“Quasi”-Steady State	no	yes, min	no		yes, min
*k*_heterogeneous-steady-state_ / s^–1^		7.80 × 10^–4^			1.54 × 10^–3^
γ_BET-Steady-State_		3.19 × 10^–8^			8.43 × 10^–8^

PBX_V_ shows a total adsorption of 6.28 × 10^12^ # molecule/cm^2^ and no “quasi”-steady-state uptake. The uptake is 7.75-fold increased to 4.87 × 10^13^ # molecule/cm^2^ for PBX_UV_, which also shows a slight “quasi”-steady-state uptake, corresponding to a faster bulk-transport phenomenon compared to the virgin state. PAXA_V_ displays an initial adsorption of 1.6 × 10^13^ # molecule/cm^2^ and also no “quasi”-steady-state, whereas PAXA_UV_ displays a more significant “quasi”-steady-state and uptake at 9.48 × 10^13^ # molecule/cm^2^, which is 5.93 times higher than PAXA_V_. PAXA_V_ also shows a 2.55-times higher H_2_O molecule reactivity than PBX_V_, whereas the difference between PAXA_UV_ and PBX_UV_ reduces by a factor of 1.95-times. Both polymers show in their UV-degraded states a new “quasi”-steady-state uptake, which is used to calculate the characteristic rate constants *k*_het-StSt_ and γ_BET-StSt_. Both of these values are higher for PAXA_UV_ compared to PBX_UV_. Table 3 discloses the increase or decrease of gas molecule uptake per SSA of PBX and PAXA upon UV degradation.

**Table 3:**
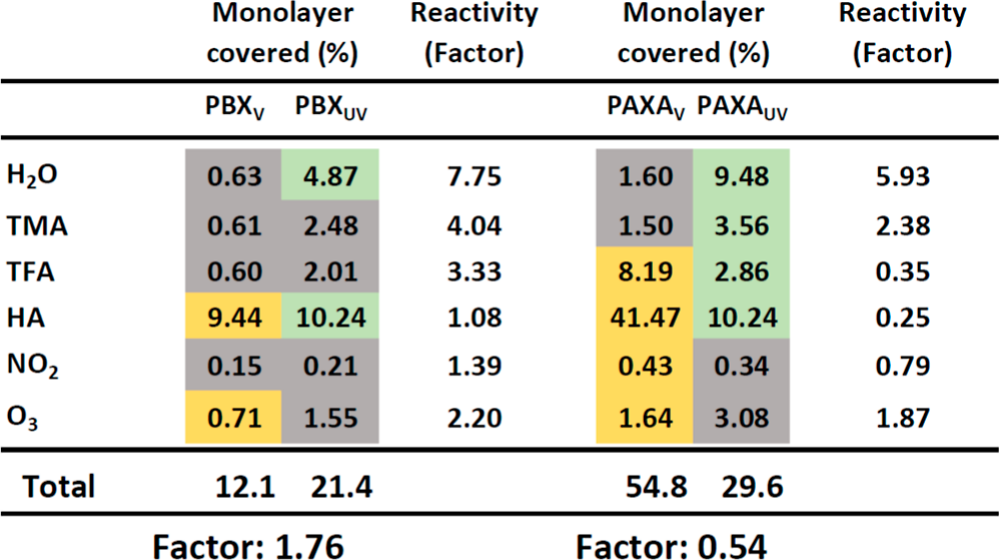
Summary of Degradation-Induced Reactivity/Uptake Changes and Surface Coverage of Probe Gas-Specific SFGs in Virgin and UV-Degradation States^a^

a Gray highlighted boxes indicate that the sample did not show loss owing to bulk transport, whereas yellow and green boxes indicate an uptake in virgin state and gas molecule bulk transport after UV degradation, respectively.

UV degradation increases the reactivity for each probe gas in the case of PBX, whereas PAXA_UV_ shows increases only for H_2_O, TMA, and O_3_. SFGs reacting with HA make up the largest fraction relative to both nondegraded states. The area of reactive sites for PBX nearly doubles (1.76), while it decreases to half for PAXA (0.54) after being degraded by UV light. UV degradation increased *k*_het-StSt_ and γ_BET-StSt_ for PAXA_UV_, when allowing bulk transport for TMA molecules and slight increases for TFA, while also showing an increasing behavior for HA. In any case, except for PAXA_UV_ + HA, PAXA shows higher values in reactivity in comparison to PBX. Detailed numerical data for each sample may be found in Table S1.

**Figure 3:**
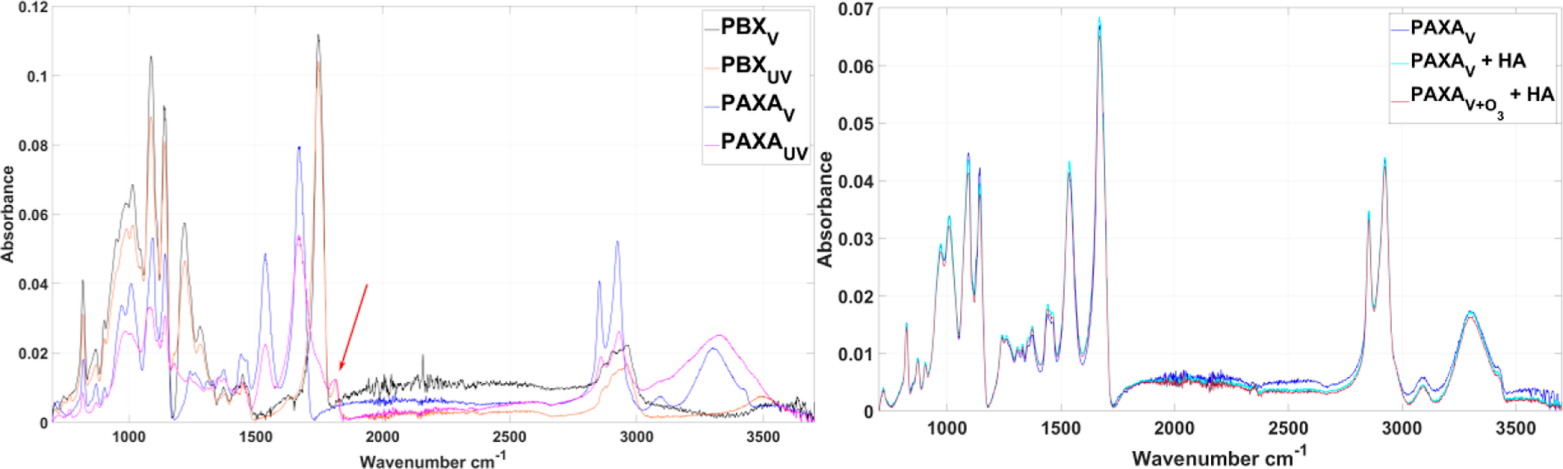
ATR-FTIR spectra of PBX_V_, PBX_UV_, PAXA_V_, and PAXA_UV_ (left) with a common peak at 1814 cm^–1^ (indicated by a red arrow), signaling novel carbonyl containing molecules for both polymers upon UV degradation (PBX_UV_ and PAXA_UV_). Right: PAXA_V_ (dark blue line), PAXA_V_ after being exposed to HA (PAXA_V_ + HA, light blue line), and O_3_ pre-exposed PAXA_V_ after consecutive HA exposure (PAXA_V+O3_+HA, brown line). Each degradation state shows identical absorbance peaks while only slight variation in absorbance intensity, likely due to changes of material packing on top of the ATR-FTIR crystal.

In the short-term degradation experiments with O_3_ as a strong oxidizing agent, the samples were initially exposed to a controlled O_3_ flow using the identical KFR setup. Following this, they were directly exposed to HA without venting the sample chamber (Table S1). The O_3_ molecule uptake as displayed in Table 3 was more pronounced for the solar simulator treated case for PBX and PAXA and increased more significantly for PBX. Both polymers show a slight bulk transport mechanism in their virgin state, which is lost after UV degradation (Tables S1 and S2). PAXA_V_ shows a significant loss of HA gas molecule uptake (5-fold) after being exposed to O_3_ (Table S1). The UV-degraded polymers show no change in reactivity after additional O_3_ degradation, when being probed by HA. As shown in Table S1, PAXA_V+O3_ does not exhibit any newly formed absorbance when analyzed by ATR-FTIR, as illustrated in Figure 3.

**Figure 4:**
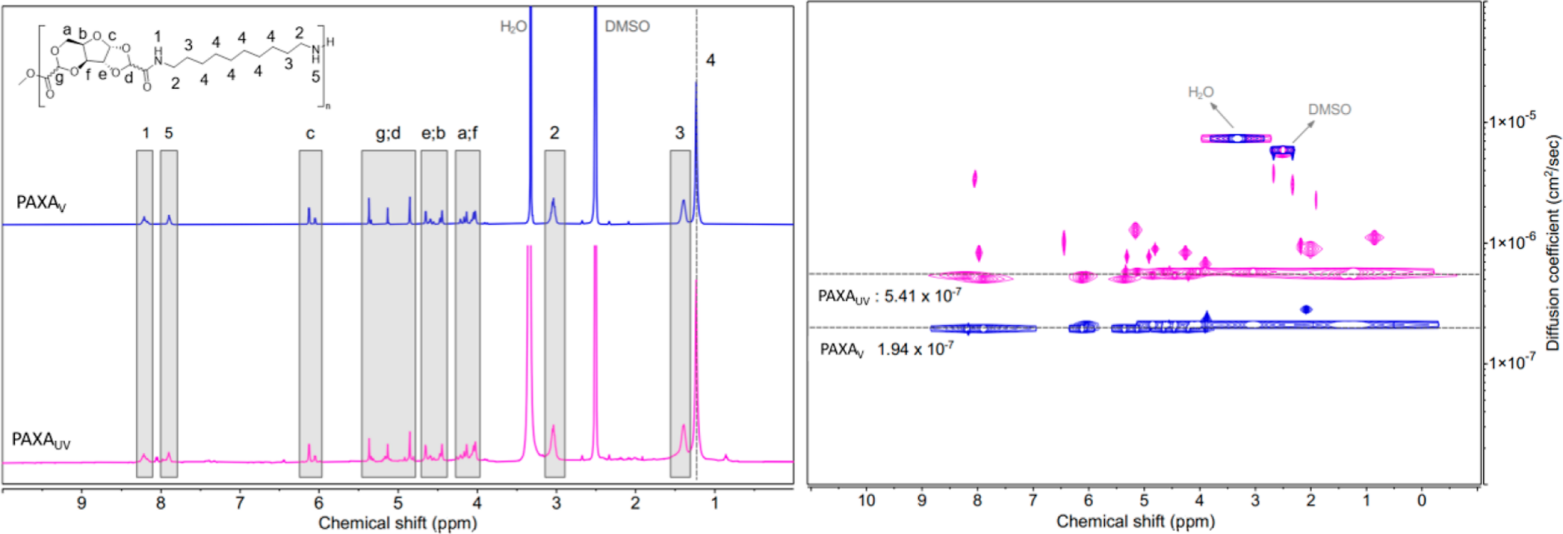
PAXA_V_ (blue) and PAXA_UV_ (magenta): ^1^H-NMR (**left**, including assignment) and 2D-DOSY-NMR (**right**): NMR experiments performed in *d*_6_-dimethylsulfoxide solvent: The black dashed lines (2D-DOSY-NMR) represent the mean diffusion rate of the proton signals associated with the polymer backbones.

Figures S12 and S13 show ATR-FTIR spectra of PAXA_V_ and PBX_UV_, respectively, after being tested with HA and exposed to HA, without any change. A general surface smoothing, loss of small particles, and discoloration were observed using SEM (Figure S14), PSA (Table S3), and UV–Vis measurements (Figure S15). Table S4 shows SEC-MALS results for PBX_V_, PBX_UV_, PAXA_V_, and PAXA_UV_, which demonstrate effective UV degradation. Corresponding chromatograms are displayed in Figure S16. Polymeric molecular weight performance indicators, tested with SEC-MALS, as presented in Table S4, are decreasing after UV degradation, e.g., the number-average molecular weight (*Mn*) reduces from 17.7 and 24.9 kg/mol to 2.4 and 3.3 kg/mol for PBX and PAXA, respectively. An increase in polydispersity was observed for both polymers, albeit it was more pronounced for PAXA_UV_. This suggests a broader range of molecular weights as a result of increased and/or uncontrolled degradation. ^1^H-NMR was utilized to check the stability of the initial polymer structure and potential differences after UV degradation. Results are presented in Figure 4 for PAXA and Figure S17 for PBX. The analysis of virgin and UV-degraded PAXA reveals structural degradation mainly affecting the polymer chain length. Long-term degradation primarily changes the polymeric structure, as specific ^1^H NMR fingerprints and signals of the original polymer chains are retained in the degraded samples. The ^1^H-NMR spectra of PAXA_UV_ show more “noise”, representing small, novel, and more mobile molecules compared to PAXA_V_, which is confirmed by the 2D-DOSY-NMR results. UV degradation impacts the polymer chain length and therefore molecular weight, while the overall structural nature of the polymers remains, when virgin- and UV-degraded states are being compared. Recorded 2D-DOSY-NMR spectra of PBX and PAXA display a more macromolecular chain length degradation, with small fragments or clusters of nuclei being created after UV exposure. The most striking one is observed at a 0.8 ppm chemical shift, visible as novel peak in the ^1^H- and 2D-DOSY-NMR spectra as a peak and cluster, respectively, for PAXA_UV_, Figure 4.

The measured proton diffusion rates of the signals belonging to the polymer chains of PAXA_UV_ and PBX_UV_ diffuse at a faster rate than their respective virgin polymer: from a diffusion rate of 3.13 × 10^–7^ cm^2^/s and 1.94 × 10^–7^ cm^2^/s to 5.00 × 10^–7^ cm^2^/s and 5.41 × 10^–7^ cm^2^/s for PBX and PAXA samples, respectively. A slower proton diffusion rate is associated with protons belonging to a large cluster of nuclei—therefore to a larger macromolecule, which supports the assumption of significant polymer chain degradation after UV degradation, which leads to smaller nuclei and a fast proton diffusion rate.^21^

## 4 Discussion

The commonly assumed increase in polymer reactivity following environmental exposure—typically attributed to an increase in SSA, as hypothesized by Vaksmaa—is not valid in this case and must be treated with caution in the future.^22^ Borgmeyer showed that long-term UV degradation experiments on PS particles led to the formation of surface cracks, while the chemical surface reactivity varied depending on the degradation factor.^7^ However, this is not supported by the current SEM results (Figure S14), nor by the decrease in BET values (Table 1).^22^

Table 3 shows increases in reactivity for all six probe gases (H_2_O > TMA > TFA > O_3_ > NO_2_ > HA) when probed on PBX_UV_ while dropping to only 26.4% of its virgin BET-value (Table 1). PAXA_UV_, in contrast, presents complex behavior (H_2_O > TMA > O_3_ > NO_2_ > TFA > HA), dropping to 39.6% of its virgin BET-value, with decreasing uptakes for NO_2_, TFA, and HA.^7^ The comparison between PBX_V_ and PAXA_V_ reveals a higher uptake for PAXA_V_ for all probe gases (TFA > HA > NO_2_ > H_2_O > TMA > O_3_), while after UV degradation (O_3_ > H_2_O > NO_2_ > TMA > TFA > HA), the uptake reactivities significantly converge. The nonweighted approximate monolayer coverage of the polymer surface highlights an increase in reactivity for PBX (from 12.1% to 21.4%) and a decrease for PAXA (from 54.8% to 29.6%) following UV degradation (Table 3). The results highlight on one side the different nature of the polymers and consequently different uptake characteristics in their virgin states while also pinpointing changes in reactivities upon varying long-term UV degradation. PAXA_UV_ shows an increase in mass transport *k*_het-StSt_ for H_2_O, TMA, TFA, and HA, while decreases were observed for NO_2_ and O_3_ (Table 3), which may be a hint at the pronounced structural degradation, changing and increasing the diversity of bulk molecules compared to PBX_UV_ (Figures 4, S17). PBX_V_ shows that *k*_het-StSt_ increases only for H_2_O and HA, signaling a lower diffusion rate into the bulk, probably owing to structural features.

The water vapor barrier of a polymer alongside the oxygen barrier properties are two of the most important material properties and of significant interest to industry and research,^23^ which may be gauged by the molecular interaction of the polymer SFGs with H_2_O as a probe gas.^24^ Uptake characteristics may act as an indicator of the material reactivity when exposed to humidity, which is an unambiguous parameter for any real-life application and includes all potential end-of-life scenarios.^25,26^ H_2_O affinity is chemically important not only in regards to food safety, e.g.,^27,28^ but also when considering degradation mechanisms with special focus on microbial polymer bioavailability, which partly depends on the initial biotic/abiotic hydrolysis step.^6,22^ The probe gas molecule uptake goes beyond sole surface adsorption, as UV-induced changes in bulk transport mechanisms were observed for PBX_UV_ and PAXA_UV_ when probed by H_2_O (Table 2). Both samples exhibit a new “quasi”-steady-state in the uptake of H_2_O. In (accelerated) H_2_O-degradation tests performed by Manker,^16^ PBX_V_ dissolves within days which may be considered fast, pointing to a high water affinity. The main driver here is hydrolysis and an autocatalytic degradation due to the release of DGAX and inherently acidification of the water phase.^16^ The higher initial reactivity of PAXA_V_ may be explained by the presence of a N–H bond and dipole, which acts as an H-bond donor and is not present in PBX. For PAXA_UV_, this particular absorbance band is intensified according to ATR-FTIR data (3303 cm^–1^, Figure S11), which may explain the increased uptake of H_2_O molecules due to its preference of forming hydrogen bonds.^29^

When H_2_O was used as a probe gas, PAXA_UV_ shows higher values of *k*_het-StSt_ and γ_BET-StSt_ than PBX_UV_, while the difference between total molecules taken up converges from 2.55 to 1.95 between the virgin and UV degradation states (Table S3). This suggests a loss of SFGs in PAXA_UV_ while exhibiting increased reactivity for mass transport into the bulk. This may be attributed to alterations in the molecular structure.

The increase in weak or strong acidic SFGs reacting with TMA is potentially new i-COOH-FGs caused by hydrolysis or oxidation and following buildup of oxygen-containing SFGs for PBX_UV_ and PAXA_UV_, while interfacial polymer hydrolysis will result in its original (acidic) monomers. Acetal hydrolysis is a second option for both polymers, leading first to a hemiacetal, followed by the formation of an aldehyde and an alcohol. PBX degradation through hydrolysis will lead to an acid and an alcohol, while PAXA degradation will lead to an acid and an amine, both functionalities support the increase in TMA uptake. The surface acidity probed by TMA increases for both polymers after UV degradation, while PBX_UV_ shows a more pronounced reactivity, as seen before with PS.^7^ PAXA_UV_ shows the only and significant bulk mass transfer for TMA, signaling that acidic-SFGs have been formed and sufficient diffusion is enabled compared to PBX_UV_, underlined by *k*_het-StSt_ (Table S1). Figure 3 shows an absorbance band at 1814 cm^–1^, signaling novel carbonyl containing molecules for both UV-degraded polymers, which highlight common degradation mechanisms of both polymers but also an expected acidification process, which may translate into an increased TMA reactivity, in case diffusion is possible. This is supported by the marginal decrease in TMA uptake compared to the significant loss in SFGs, when probed with HA and TFA for PAXA_UV_ (Table 3). However, the possible formation of acidic i–OH groups as a candidate for interaction with TMA as reported by Diebold on TiO_2_ may not be discarded.^30^ The increase in i-COOH groups for both PBX_UV_ and PAXA_UV_ also increases the hydrophilicity of the samples confirmed by the strong increase in H_2_O molecule uptake.

The uptake of O_3_-molecules for both polymers approximately doubled after UV degradation (Table S1), while both virgin polymers also display a bulk, albeit slow, mass transport rate, which is lost after UV degradation. The specific bulk mass transport parameter changes in the case of PAXA for each individual probe gas, except for the short-term O_3_ treatment (Table S1). O_3_ shows low and NO_2_ the lowest reactivity for both polymers, signaling the highly oxidized interface of the virgin state of both polymers.^16,17^ However, uptake changes were more significant for virgin polymers, which may be related to the less perturbed and more pristine molecular bulk structure of the individual polymer.

The weaker oxidizer NO_2_ shows the lowest level of reactivity with all four UV degradation states and also the lowest *k*_het-StSt_ with PAXA_V_ (Table 1). Significant uptake of NO_2_ on virgin polyester, as claimed by Clark on propylene carbonate, is improbable.^31^ In line with previous results on PS, the polymers investigated using KFR exhibit distinct behavior when exposed to the probe gases, likely due to differences in their polymeric nature.

The amount of reactive SFGs probed by TFA shows a significant increase for PBX_UV_ in contrast to a decrease for PAXA_UV_ (Table 3). PAXA_V_ shows a 13.56-fold higher reactivity to PBX_V_, which reduces to 1.42-fold after UV degradation. A similar decrease in SFGs may be observed when PAXA_UV_ was tested with HA, in which case PBX_UV_ showed a 4.4-fold higher uptake. The authors conclude a significant increase in basicity for PBX_UV_, but PAXA shows in both interpolymer degradation states the higher total number of TFA molecules taken up, but in contrast a smaller increase upon aging.

I–OH groups probed by HA marginally increase in number for PBX, while they decrease significantly for PAXA, together with a significant increase in hydrophilicity for both UV-degraded polymers. PAXA_UV_ shows an equal HA molecule uptake compared to PBX_UV_, while only half of *k*_het-StSt_ is present (Table 3). Both values were greater when comparing the virgin states, where PBX_UV_ also showed the highest total molecule uptake for any probe gas (Table 1). PAXA displays a loss of i–OH-FGs, presumably owing to an etching reaction, upon HA-probed UV degradation, since *k*_het-StSt_ experiences only a minor increase (Table S1). The higher reactivity to HA is expressed by the fact that PBX_UV_ shows higher initial uptake kinetics at equal overall uptake. This may suggest a severe loss of i–OH-FGs on the surface- and bulk-level for PAXA_UV_ (Table S1), originating from long-term UV degradation.

PBX_V_ and PBX_V+O3_ show identical uptake reactions when probed with HA, while PAXA_V+O3_ shows only 21% of the reactivity compared to that of PAXA_V_ (Table S1). PBX_UV_ and PAXA_UV_ do not reveal any change upon pre/post O_3_ degradation in HA molecule reactivity, nor do they show a significant change in steady-state behavior for any of the four polymers in contrast to PAXA_V_ (Table S1). The authors conclude that when probed with HA, long-term UV and short-term O_3_ degradation may have similar effects on identical SFGs, solely depending on the nature of the polymer. Short-term O_3_-induced degradation has a severe effect on the interfacial reactivity, but to a lesser extent on the bulk transport of PAXA_V_. The increase in *k*_het-StSt_ is the sole effect on PBX_V_. The experiment also showcases the exclusive nature of using aqueous O_3_ as a degradation agent. Table S1 displays smaller *k*_het-StSt_ and γ_BET-StST_ values for both virgin polymers, compared to the UV-degraded counterparts, due to bulk degradation (Figure 4). In fact, PBX_UV_ and PBX_UV+O3_ display the highest mass transport values, when probed with HA (Table S1). UV exposure decreases SFGs as well as interfacial transport into the polymer bulk slightly. Since the O_3_-exposure was around 20 min and bulk properties did not change, the authors conclude that mainly interfacial-FGs were exclusively degraded. PAXA_V_ shows a new “quasi”-steady-state but unlike UV degradation, O_3_ did not change any steady-state uptake, suggesting O_3_ degradation to be less destructive for the bulk than UV degradation.

In general, UV exposure creates new absorbance bands and an overall loss in intensity. Nevertheless, it cannot provide insight into surface-exclusive interfacial changes on a molecular level, especially following short-term degradation, as demonstrated with the O_3_-treated PAXA sample (Figures 3 and S13).^7,9^ The irregular changes in bulk transport for PAXA_UV_ suggest pronounced changes upon long-term UV degradation, which are likely based on molecular polymer chain degradation, which can be justified by ^1^H- and 2D-DOSY-NMR results. The quantifiable changes in *k*_het-StSt_ and γ_BET-StSt_ document the selectivity and differentiation of the KFR experimental method, with regard to (polymer) surface and bulk reactivity. This differentiation is important for a relatively short time scale of up to 60 min upon SC opening as presented here, but even more significant for long-term reactivity under realistic conditions, since PAXA_UV_ may slowly but steadily “consume” reactive molecules, until complete saturation of the SFGs.

Vaksmaa reports significant chemical reactivity changes in the early stages of a long-term degradation process before a reactivity-“plateau” is attained,^22^ signaling a small but steady increase in reactivity, potentially due to increases in bulk transport over time, as experienced here but slower. The molecular chain scission and degradation of bulk polymers (Figure 4) may be the reason for the changes in interfacial reactivity and mass transport to the bulk since the polymer reactivities ultimately depend on its molecular structure as presented here. Surface functionality may be evaluated using long- and short-term degradation conditions. This may explain why degraded polymers exhibit increased overall reactivity, particularly when subjected to prolonged exposure under varying and successive conditions.^7^ In general, an increased degradation results in more polymer bond breakages, generating a higher number of bulk reactive FGs, assuming that diffusion into the bulk becomes gradually more feasible. Two counteracting mechanisms compete according to the presented results. The SSA decreases, while new FGs form as a result of molecular degradation occurring within the bulk of the material. The 2D-DOSY experiments, observations of low molecular weight compounds and higher polydiversity index in the case of PAXA_UV_ (Figure 4), may indicate “reactive artifacts”, which lead to the increased/decreased reactivity, also reported by Ainali.^32^ Individually, SEC-MALS and NMR results may lead to different conclusions compared to when both methods are combined. The quantified intrinsic polymer properties (Table S4) suggest a uniform degradation state. However, NMR, especially 2D-DOSY, analysis reveals the details of the actual degradation process, indicating increased degradation of PAXA_UV_ and consequently the formation of unidentified smaller molecules. Contrary to expectations, PAXA demonstrates an unanticipated decline in surface reactivity, accompanied by significantly altered diffusion properties during degradation (Tables 3, S2) when compared to PS.^7^ A comparison to PBX, due to shorter UV degradation duration, was omitted. In both cases, a time-series may explore additional details about the degradation pathways.

Following the presented material degradation, both polymers share the identical copolymer DGAX. It makes up 61% of the molecular weight of PAXA and 75% of PBX based on their chemical structure (Figure 1). The higher intrinsic bond-stability of polyamides, tested with SEC-MALS, makes PAXA more resistant to UV degradation compared to PBX.^16,17^ Manker shows that chemical degradation affects the polymer stability, while DGAX withstands harsh conditions at first,^16^ which is similar to the presented case of PBX_UV_. The easily hydrolyzable nature of PBX was verified by the solubilization of PBX_SS_ when exposed for >100 h to UV light as a follow-up reaction to smelting (Figures S3 and S14).^16^^1^H NMR displays an essentially undegraded molecular structure, while intrinsic material properties and stability are completely degraded. PBX exhibits only permanent dipole–dipole interactions, whereas PAXA additionally participates in intermolecular hydrogen bonding and permanent dipole–dipole interactions due to polar C=O and C–N groups. Both polymers show van der Waals interactions, making PAXA the more resistant polymer confirmed by SEC-MALS.

Experiments show the complete degradation of PBX in industrial compost processes (aerobic or/and anaerobic) after 3 weeks, whereas PAXA did not show disintegration.^18^ A second UV experiment involved strips of 10 × 50 mm in length and 1 mm thickness of PBX and PAXA that showed an identical change in color followed by solubilization of PBX after 6 days while the flexibility of PAXA (25 days of exposure) turned into brittleness, indicating cross-linking between polymer chains.

Results presented throughout highlight the significance of applying a more sensitive molecular titration approach compared to bulk studies, together with tests on the initial and degradation-induced reactivities of polymeric surfaces. In addition, it also pleads for more time-series measurements in the short-term exposure regime. Most degradation mechanisms are not known yet for PBX/PAXA owing to the lack of investigations in this regard. The NMR and SEC-MALS results presented here confirm the bulk degradation of both polymers whose mechanisms are as yet unknown and highlight the approach of analyzing an in-house-developed polymer. Commonly used ATR-FTIR shows clear limitations with respect to surface sensitivity, which are confirmed here (Figures 3, S13).^7^ The interpretation of ATR-FTIR in relation to KFR results is fraught with difficulties; therefore, the authors limit themselves to a meta-assessment of the collected results with focus on change in SFGs pre/post UV and/or O_3_ degradation, also due to a lack of data. Still, the authors are aware and remind the reader that the KFR operates at high-vacuum testing conditions for the removal of most polymer-adsorbed H_2_O which might also affect the organic material and therefore transferability of measured reactivities to real applications.

Industries worldwide are looking for conventional polymer substitutes and greener alternatives, either biobased and/or (bio)degradable.^33^ The polymers investigated herein are the first sustainable polyesters and polyamides based on largely unmodified functionalized lignocellulosic sugars, which have been subjected to a degradation study investigating the change in interfacial reactivity under environmentally relevant UV and O_3_ conditions. The material samples were examined using standard “surface” analysis techniques to assess their sensitivity in comparison to KFR results. The relatively short time scale reveals the initial reactivity of the polymer interface, while longer exposure periods might disclose relatively slow transport processes into the 3D-bulk structure of the sample. The present work emphasizes the benefits of conducting polymer analysis at an early development stage, specifically during the research and development phase when the polymers remain chemically unmodified. The results look promising in regards to the impact of isolated artificial degradation, its effect on the surface functionality of (bio)polymers, and the changes in interfacial reactivity, which can be chemically identified and quantified using the KFR. The presented results also underscore the current limited understanding of degradation mechanisms concerning the SSA and resulting reactivity. The limitations of commonly applied techniques are made apparent in regards to characterizing the first contact and attachment point of materials at the interface. Compared to previously communicated findings performed on PS using the KFR, the interfacial reactivity shows significant differences before and after experiencing degradation (Table 3). This may also result from the unknown chemical composition of the investigated PS and degradation conditions^27,34^ but is primarily attributable to the distinct polymeric nature of PS and the differences to the here presented samples, such as simplicity of monomer styrene and a more complex copolymer structure of PBX and PAXA.

In conclusion, using a chemical surface-sensitive titration approach looks like a reasonable analytical approach to assess every conceivable degradation state, especially after short-term degradation. The authors therefore claim that once a polymer is chemically fully characterized in the laboratory, one can understand and predict its reactivity in different environments following different degradation mechanisms. Suitably performed laboratory experiments and degradation scenarios could fully define and disclose changes in reactivity upon isolated or combined environmental degradation factors. These changes in reactivity lead to varying behaviors when exposed to reactive substances or organisms,^22,35^ which are again heavily influenced by environmental conditions (e.g., low/high pH, low/high temperature, etc.), as shown by Borgmeyer.^7^ The current findings indicate that quantifying severe polymer degradation does not necessarily result in substantial increases in SSA and, consequently, polymer reactivity. Hypotheses presented in literature need verification, and the authors point out that a generalization of degradation patterns and assumptions based on increases/decreases in reactivity derived from chemical reactions without presenting surface sensitive analysis and proof are risky.^22^ This work offers a preliminary exploration into the potential of integrating standard chemical engineering methods used in polymer research with physicochemical principles to elucidate the degradation process at both the molecular and interfacial levels.

### 4.1 Environmental Implications and Perspectives and Outlook for Future Knudsen Flow Reactor Studies

The authors would like to highlight their assumption of a chemical plateau being build up or present in any degradation state determined by its surrounding environment(s). This saturation effect may also be experienced by Vaksmaa in wet-phase experiments but were potentially wrongly interpreted.^22^ The reactivity might increase or shift, maybe alongside increases/decreases in SSA, but the materials’ reactivity is set by its chemistry and surface, which is covered and defined by reactive and space-dependent SFGs. Once the chemistry and its state-dependent reactivity are understood and normalized to a specific reactivity per unit surface area, the material’s environmental persistence may be evaluated and either calculated or predicted. This is of the highest relevance in the field of (bio)degradable polymers or chemistry to evaluate the degradability or “constant reactivity” under realistic conditions. Only a wholesome analysis of polymer properties in combination with surface sensitive analytics will disclose its unique degradation characteristics; a generalization, unproven claims, assumptions, and theories is not effective. Given this outcome on three polymers, the authors are encouraged in their belief that the buildup of a “chemical-(topological)-reactivity-map”, containing nowadays most relevant (bio)plastics, must be the scope of future studies. To be able to fully understand the impacts plastics (particles) have on the environment, researchers must understand their degradation mechanisms and consequently surface reactivity at their various degradation states. As often shown, degradation states influence, if not determine, the likeliness of chemical reactiveness and responsiveness of polymers. Undoubtedly, the amount of polymer particles ending up in the environment will only increase in the foreseeable future,^1^ and current knowledge compared to here presented results suggest: little is known about reactivity changes upon (a)biotic environmental exposure.

Based on existing knowledge and the findings presented here, the authors propose three possible pathways resulting from degradation, illustrated in Figure S19. The presented uptake and transport kinetics are important since they may determine long-term reactivity, risk, and persistence of polymers and chemicals attached to them.

Moving on, researchers must focus on the unknown: short-term degradation and its implications. As HA probe gas results suggest: a month of O_3_-exposure has a similar effect as several years of UV degradation, mimicked in a laboratory environment, in regards to i–OH SFGs. Here, one unknown remains: how do current methods, protocols, and approaches allow necessary sensitivity to identify experienced changes. Kinetic measurements of uptake coefficients will help to differentiate between long- and short-term degradation mechanisms, which are required to understand both behaviors of plastic particles in various environments.

## Supporting Information Available

Detailed Information about Polymer Processing, Setup, Equipment, Methods and Protocols used; configured and modified KFR settings and workflows, and compiled and analyzed results (PDF)
