# Supplementary material for: Characterization of Biobased Polymers at the Gas–Solid Interface—Analysis of Surface and Bulk Properties during Artificial Degradation
Source: Environ Sci Technol. 2025 Apr 19;59(16):7845–54. doi: 10.1021/acs.est.4c10925 (PMC12044678; doi:10.1021/acs.est.4c10925)
Supplement: Supplementary file 1 — es4c10925_si_001.pdf [file es4c10925_si_001.pdf]

Supporting Information (SI)

Characterization of Biobased Polymers at the Gas-Solid Interface – Analysis of Surface and Bulk Properties during Artificial Degradation

T. Borgmeyer [1], Y. Kupper [2], M.J. Rossi [1], J. S. Luterbacher [2], C. Ludwig [1,3]*

[1] École Polytechnique Fédérale de Lausanne (EPFL), GR-LUD, School of Architecture, Civil and Environmental Engineering (ENAC IIE), Station 6, CH-1015 Lausanne, Switzerland; tobias.borgmeyer@epfl.ch; michel.rossi@epfl.ch

[2] École Polytechnique Fédérale de Lausanne (EPFL), Laboratory of Sustainable and Catalytic Processing (LPDC), Institute of Chemical Sciences and Engineering (ISIC), Station 6, CH-1015 Lausanne, Switzerland; yildiz.kupper@epfl.ch; jeremy.luterbacher@epfl.ch

[3] Paul Scherrer Institute (PSI), Center for Energy and Environmental Sciences, CH-5232 Villigen PSI, Switzerland; christian.ludwig@psi.ch

* Corresponding author

<u>Summary:</u> 25 pages, 4 tables, 19 figures

1. Material Processing, Methods and Protocols

4s-PBX and 4s-PAXA 10

PBX

PBX was dried overnight in a vacuum oven at 60°C before undergoing compression molding. The dried PBX was melt-processed at 140°C in a 6 cm × 14 cm mold under a 10 kN load for 20 min, with heating and cooling ramps of 10 °C/min, yielding films of 0.5 mm thickness. Films were homogenized using a XXX Further methodological details can be found in Manker et al., 2022.

PAXA

The 4s-PAXA 10 samples were compression-molded using a Lauffer vacuum-assisted hot press. Prior to molding, the polyamide was dried at 80°C for 24 hours. The dried material was then hot-pressed for 10 minutes under vacuum conditions at 220–250°C, depending on the polymer's glass transition temperature (Tg), applying a pressure of 60 N/cm². Films were homogenized using a XXX Additional methodological details are provided in Manker et al., 2024.

Polymers were then granulated using a laboratory grinder and supplied as inhomogeneous pieces of 1-5mm in size.

Cryogenic-Milling

A high surface to volume ratio was the goal of the cryo-milling sample preparation.
Cryogenic milling is required since mechanical treatment and impact forces create heat, which would alter polymer material properties and potentially lead to thermal degradation and melting.
Milling conditions involved three consecutive cycles, with changing steel ball diameter and number: A mass of 3 g of granular sample was added to the 50 ml crucible and a 20 mm Ø steel ball was used in the first milling step. Pre-Cooling conditions were set to 180 s at a frequency of 5 Hz horizontal oscillation speed. Milling cycle parameters were set to five cycles of 180 s at 30 Hz with intermittent milling 'breaks' of 30 s at 5 Hz. For the second cycle the 20 mm steel ball was replaced by twelve Ø 10 mm steel balls, for the third run a total of six 10 mm steel balls were used. A homogeneous powder of plastic particles for surface to volume ratio maximization was the goal. Plastic powders were stored in glass vials in a desiccator connected to a membrane pump at 100 mbar pressure.

Brunauer-Emmett-Teller (BET) Specific Surface Area (SSA) analysis

Sample tubes were cleaned with LC/MS-grade EtOH (≥99.8%, Fisher Chemical), brushed with a natural fiber pipe cleaner, blown out with filtered compressed air, and stored in an oven at 120 °C until use. Before and after filling, the tube was left at ambient temperature to cool down before weighing. The cryo-milled polymer sample was added to the sample tube using a long flat/spoon end spatula to prevent powder deposition on the sample tube wall. Each filled sampling tube was degassed at ambient temperature in a preparative line until a steady pressure value of 3 mTorr was reached. The tube was filled with inert N_2 gas and isolated from ambient air until connection to the 3 Flex system. This last step did not last longer than 30 minutes to prevent any moisture adsorption. Before starting the measurement, the sample was further evacuated to high vacuum (<10-1 mTorr) by the turbo pump of the 3-Flex to remove any physisorbed H_2O. The Brunauer-Emmett-Teller (BET) method was used to evaluate the SSA in the P/P_0 range of 0.05–0.3. Due to the low N_2 uptake and detection limit, samples were evaluated only using the P/P_0 range from 0.05 to 0.15 to receive a reasonable BET surface area plot. P_0, was the ambient pressure around 731.2 mmHg.

Particle Size Analysis (PSA)

50mg well-mixed cryo-milled plastic powder was added to 50 ml EtOH (≥99.8%, Fisher Chemical). The solution was continuously stirred at 500 rpm, a 10ml sample taken by using a 10ml graduated glass pipette, at the 25ml mark of the beaker for homogeneous, reproducible and representable sampling.

Ultraviolet (UV) solar simulator

The Black Standard temperature was set to 35°C, which was continuously monitored by using two thermocouple probes (type K) inside the UV chamber, connected to a FLUKE 52 K/J read-out unit.

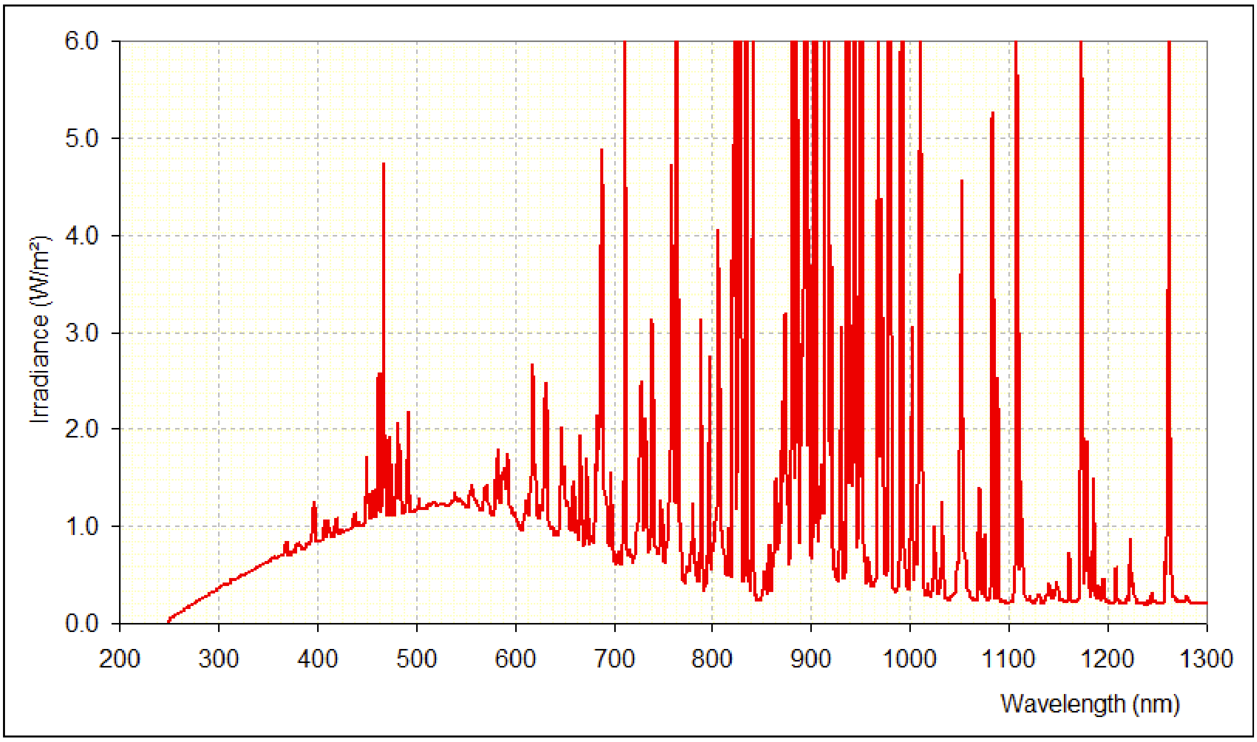

Figure S1. Emitted Spectra Lamp NXE of utilized 1700, without any filter installed. (provided by Atlas Ametek)

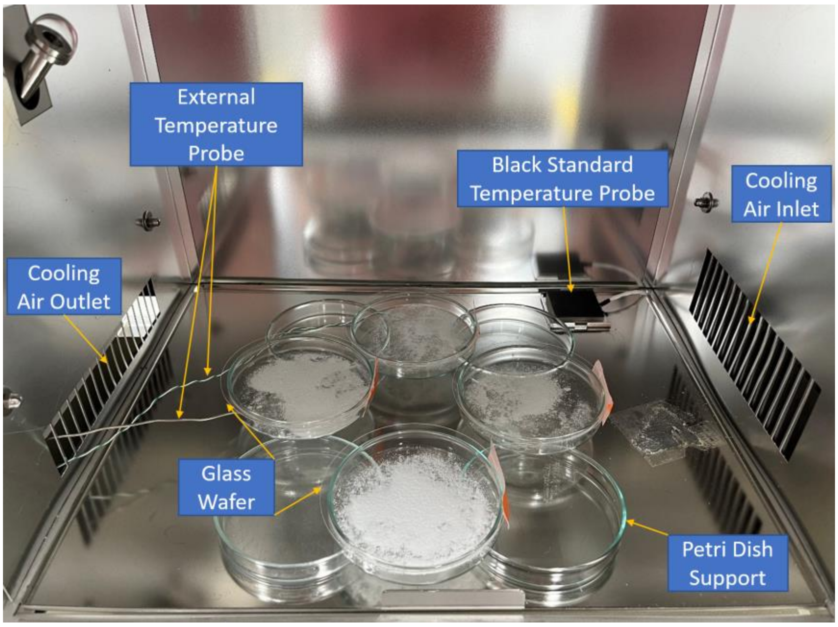

Figure S2. Solar Simulator Setup for UV degradation tests. Powders are spread inside a petri dish, while being covered by silica wafer and orange duck tape used to fix wafer to petri dish, UV source above.

Samples were placed atop of glass petri dish “supports” inside the chamber to prevent thermal degradation due to heat transfer via the steel chamber floor and assure only UV degradation. Two thermocouples were installed inside the chamber to verify air temperature won’t exceed 35 °C. Throughout the experiment, a distinct smell was noticed from either PBX and/or PAXA samples when opening the sample compartment (SC).

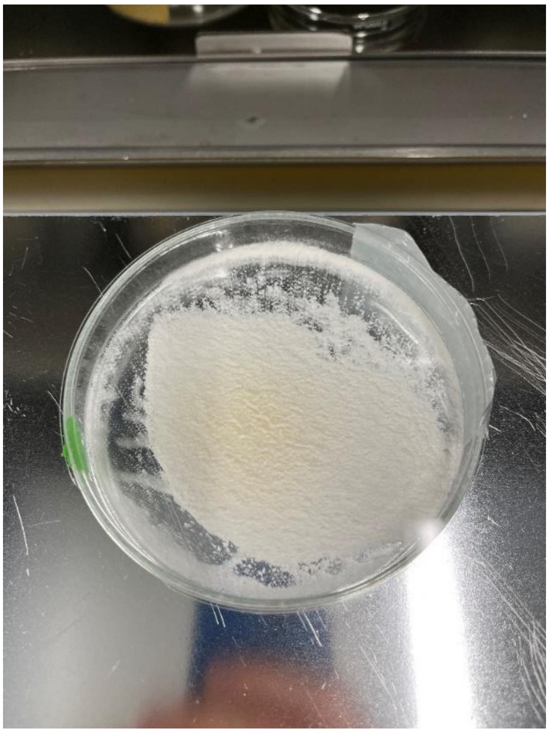

Figure S3. Discoloration of “sintered” PBX after 65 h of UV degradation

When smelting of the top layer was observed (ESI figure S3), only a loose particle fraction was recovered and used as a homogeneous sample for further analysis. The ambient temperature and bottom of the Petri dishes never exceeded 35°C, thereby preventing thermal degradation. Petri Dishes have been mixed on daily basis to allow even UV degradation.

<u>Equation 1: </u>Calculation for days of O_3 exposure

Definition: 1 ppb = $1*10^{-9}$

1 atm = $760*3.21*10^{16} = 2.44*10^{19}$

@ atm 1 ppb (#/cm^3) = $2.44*10^{-9}$

Assumption: **100 ppb** O_3 in breathing air @ atm

Flow = $6.35 * 10^{15}$ (#/s)

Dose = $6.35 * 10^{15}$ (#/s) * 20 (min) * 60 (s/min) = $7.62*10^{18}$ (#)

How many seconds under realistic conditions:

$7.62*10^{18}$ (#) = $100 * 2.44 * 10^{10} * x$ → $x = 1.56*10^{6}$ s → **<u>36.15 days</u>**

Attenuated Total Reflectance – Fourier Transformed Infrared (ATR-FTIR)

The Spectrum 2 is configured with a diamond crystal and analysis performed at an incidence angle of 50 degrees. The samples were compressed by applying a force of 80 on the software’s arbitrary pressure gauge scale. Spectra were taken between 700-3700 cm^{-1}; the generated transmittance spectra changed to absorbance, all data points reworked with the advanced ATR correction function, also correcting for the UATR diamond accessory and finished by correcting the baseline using the specific functions of the spectrum 10 software. According to the supplier, the material penetration depth of the infrared beam is around 2-4 µm, which was confirmed by calculations previously reported.[1]

Knudsen Flow Reactor (KFR)

The execution of reproducible adsorption and desorption experiments sample material was prepared and introduced into the KFR following a strict protocol: to prevent unintended degradation, samples were stored in a vacuum hose fitted desiccator, connected to a membrane pump at 100 mbar upon testing. A Pyrex® petri dish of 10.6 cm^2 horizontal geometric surface area was filled with a sample mass between 160-200 mg, using a laboratory scale, Sartorius Secura® 224-1S, placed inside the sample chamber and degassed (Ø > 20 h) until steady pressure conditions were reached. Before isolating the sample chamber, the MS was used to check for residual H_2O. Sampling gas flow was set and verified via MS at specific molecular masses before starting the uptake experiment. The uptake characteristics of the empty petri dish has been analyzed for each sampling gas and subtracted.

Scanning Electron Microscopy (SEM)

A 20 nm Au/Pd film was deposited after samples were loaded on a tape and filtered compressed air was gently used to remove all loosely spread particles.

Ultraviolet – Visible (UV-Vis) Reflectance

The UV-Vis measurements were performed in UV-Vis diffuse spectroscopy mode with integrating sphere working in reflectance mode using the UV Probe 2.7.1 software. The samples were analyzed in form of a pressed pellet in a wavelength range of 200-800 nm for semi-qualitative assessment of color change for all samples before/after light source exposure. Barium Sulfate was used as standard sample for baseline determination.

Size Exclusion Chromatography – Multi-Angle Laser Scattering (SEC-MALS)

The SEC-MALS system is equipped with a Shodex HFIP-806M Column and a Wyatt Technologies Dawn Heleos II MALS detector (λ = 663.6 nm). Agilent Technologies 1260 Infinity II pump, Agilent Technologies 1100 Series autosampler, Wyatt Technologies T-rEX refractive index detector, two PSS PFG columns, and a PSS PFG pre-column. For sample preparation, ~5mg of all samples was precisely weighed and left to solubilize overnight- to ensure full dissolution of the polymer chains, in approximately 1mL of a 5mM solution of Potassium trifluoroacetate (K-TFA) in hexafluoro isopropanol (HFIP). The masses of KTFA/HFIP solution added to each sample were measured to precisely calculate the concentration of each sample. Prior to the injection, all the samples were filtered using a 0.45 µm polytetrafluoroethylene (PTFE) syringe filter. The data were processed using the Astra 7 software. The d_n/d_c value of the PAXA-10 polyamide material was estimated on the Virgin material to be 0.210 from the RI signal by assuming 100% mass recovery to carry out the molecular weight analysis of PAXA-10 samples. The reported d_n/d_c value of 0.169 was used for PBX samples analysis. The SEC mobile phase was prepared using commercial HFIP (purchased from abcr GmbH, ABCR, AB102635-0002.00-KGM) and commercial KTFA (purchased from Chemie Brunschwig AG, Fluorochem, FLU001216-500g) and filtered on a 22µm PTFE filter.

Injection condition:

Injection volume	50µL
Mobile phase	5mM KTFA/HFIP
Flow rate	0.6mL/min
Column	Shodex HFIP-806M Column, 5 µm, 8 mm x 300 mm, 1/pk
Column temperature	Room Temperature
Analysis time	65min

Nuclear Magnetic Resonance (NMR)

The Bruker 2D DOSY NMR pulse sequence (ledbpgp2s) was used to measure diffusion coefficients. The DOSY diffusion time interval (d20) and gradient pulse length (p30) were set at 2500 ms and 100 ms, respectively, with a recycle delay (d1) of 2 s. Each 1D free induction decay had 4K complex points with 16 scans averaged. The diffusion gradients were ramped from 2% to 98% at linear increments to generate 32 increments in the diffusion dimension. DOSY NMR data processing was performed using Bruker Topspin (Bruker) and MestReNova (Mestrelab Research S.L.) software.

PBX and PAXA NMR signals: PBX - ^{1}H NMR (400 MHz, DMSO) δ 6.14 (dd, J = 28.5, 3.8 Hz, 1H), 5.68 – 5.10 (m, 2H), 4.65 (d, J = 3.8 Hz, 1H), 4.58 – 4.49 (m, 1H), 4.26 – 4.02 (m, 7H), 1.74 – 1.58 (m, 4H). PAXA- ^{1}H NMR (400 MHz, DMSO) δ 8.20 (q, J = 8.6 Hz, 1H), 7.89 (d, J = 5.8 Hz, 1H), 6.12 (d, J = 3.8 Hz, 1H), 5.41 – 4.82 (m, 2H), 4.68 – 4.40 (m, 2H), 4.25 – 3.99 (m, 3H), 3.05 (q, J = 6.7 Hz, 4H), 1.39 (s, 4H), 1.24 (s, 12H).

2. Results

KFR workflow

Figure S4 gives an overview of the most important KFR features which are already reported in Setyan et al., 2009, Mirghaffari et al., 2021, and Iannarelli et al., 2022, among others. Figure S4 presents a simplified schematic of the experimental setup for the KFR, as utilized throughout this study. In this configuration, the system is connected to a storage vessel containing H_2O probe gas, which passes through a Teflon coated vacuum line, the reactor, where it passes by the sample material before being detected by a mass spectrometer (MS).

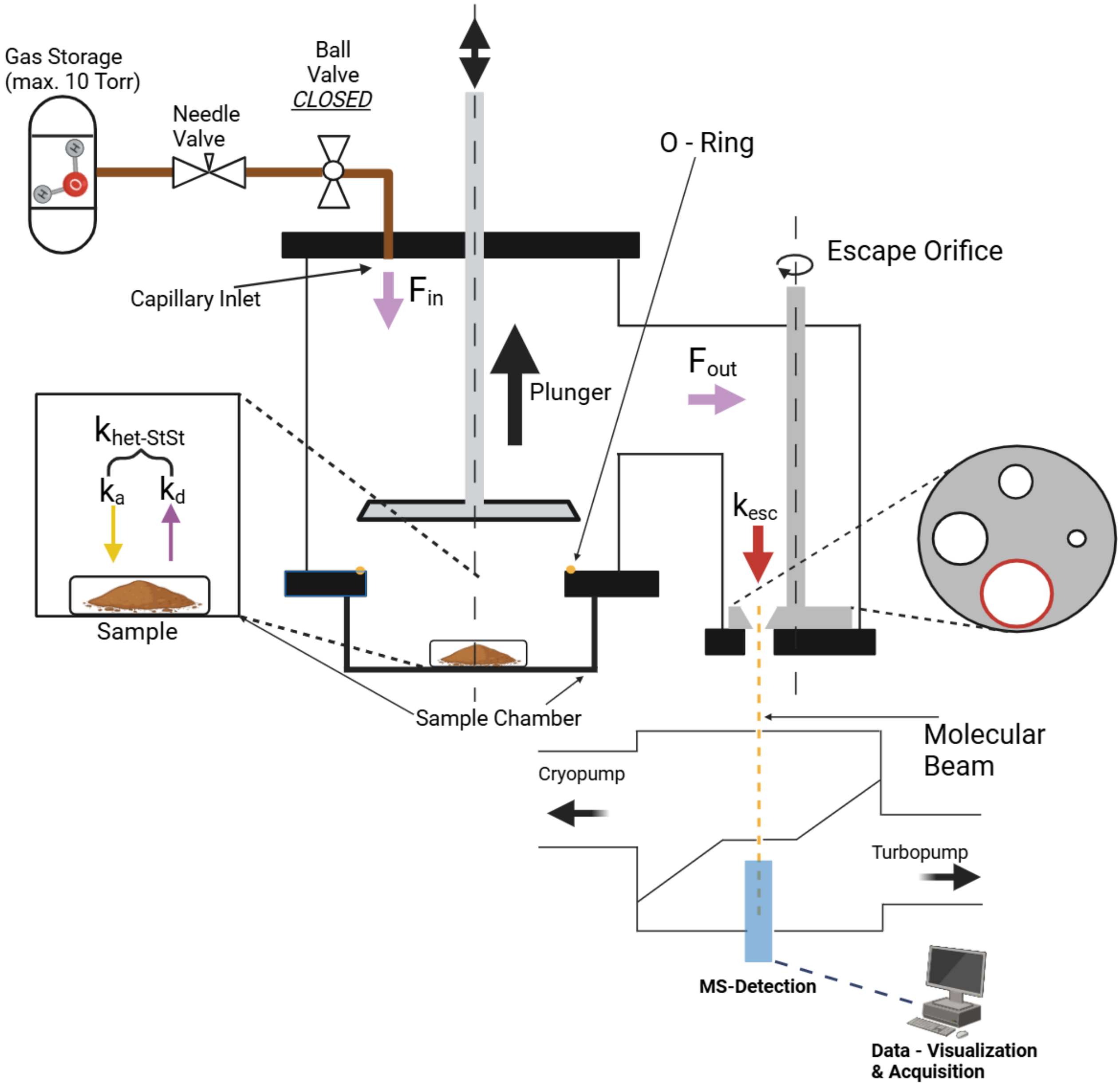

Figure S4. Schematic drawing of the KFR connected to an H_2O probe gas supply, illustrating the manual plunger mechanism for opening and closing the SC, and the off-center escape orifice directing a focused molecular beam into the two-chamber reactor. **Mode**: Overnight pumping, sample degassing @ 14 mm escape orifice and ball valve closed.

The key components of the KFR system consist of a probe gas storage vessel with connectors, a probe gas inlet line, and a two-chamber reactor. This reactor is equipped with a plunger that isolates the SC from the plenum, utilizing a Viton® O-ring to ensure a leak-proof seal. The system features an adjustable, off-centered escape orifice with nominal diameters of 1, 4, 8, and 14 mm, corresponding to specific outlet surface areas, which are connected to the vacuum chamber containing the mass spectrometer that filters and samples the molecular beam, as illustrated in Figure S4.

The sample containing petri dish is added to the SC of the KFR, the plunger lifted and the whole system pumped at 14 mm escape orifice diameter, until the sample-physiosorbed H_2O is desorbed and a constant pressure has established as displayed in Figure S4. This process takes approximately 20h for the presently investigated polymers and usually was performed overnight. Before closing the SC, the H_2O signal corresponding to the flow of molecules was checked and verified using the MS as a double safeguard for the

smooth and unbiased execution of the experiment, displayed in Figure S5, where the gas flow is started at label C.

Once the SC is closed (isolated) and the data acquisition started a steady flow of probe gas molecules can be set by opening the valves of the vacuum lines. The flow rate is adjusted using a needle valve and the rate is solely determined by the rate of molecules effusing across the escape orifice into the two-chamber reactor via the molecular beam (k_{esc}). Once a steady-state flow (F_{in}) has been established at the 14 mm orifice size, it is changed before an uptake experiment to 1 mm orifice and the user is ready to start the experiment once steady-state flow has once again been established. Figure S5, label E to label J are connected by a drawn yellow line, which represents the steady-flow of molecules (F_{out}). Slight increases might indicate a change in ambient laboratory temperature. This takes longer for “sticky” gases such as hydroxylamine (HA) and trifluoracetic acid (TFA) and is a fast process of less than 10 min for gases such as H_2O.

Upon opening of the SC, one may observe the loss in probe gas molecules on the Labview software (start of part E, Figure S5). From this point on, two competing mechanisms may take place on the sample: the rate of probe gas molecule adsorbing (k_a) and the rate of desorbing (k_d) to/from the sample, which both add up to the polymer-steady-state specific heterogeneous rate coefficient of uptake ($k_{het\text{-}StSt}$ in s^{-1}) and uptake probability per SSA ($\gamma_{BET\text{-}StSt}$), as explained by Mirghaffari et al., 2021. After either the previous or a new “quasi”-steady-state has established, the plunger is manually lowered once again to close the SC and one can observe the increase to the initial steady-state, in case a new one has formed, since competing reactions are no longer possible, Figure S5, part I to J. The molecular flow rate is determined by measuring the decrease in the probe gas pressure over a specified time interval. Throughout the experiment, the background signal of H_2O was continuously monitored, as indicated by the signal around 2.2 V at 9 500 datapoints, 12 500 datapoints, and similar intervals. This procedure was implemented to ensure the stability of the NH_2OH probe gas flow and its ratio to H_2O. In previous experiments, NH_2OH crystals exhibited decomposition and water vapor accumulation due to their hygroscopic nature, which compromised the accuracy of the flow measurements. Therefore, regular NH_2OH:H_2O ratio checks were introduced as a precautionary measure to ensure the reliability, comparability, and transferability of the results. In case the sample is not fully saturated the SC is opened another two to three of times at same probe gas conditions and previous KFR manipulation steps, until full saturation of SFGs is “achieved” or a clear new “quasi”-steady-state has been established, signaling a slow transport mechanism into the bulk as displayed in Figure S5, part E to F, G to H and I to J.

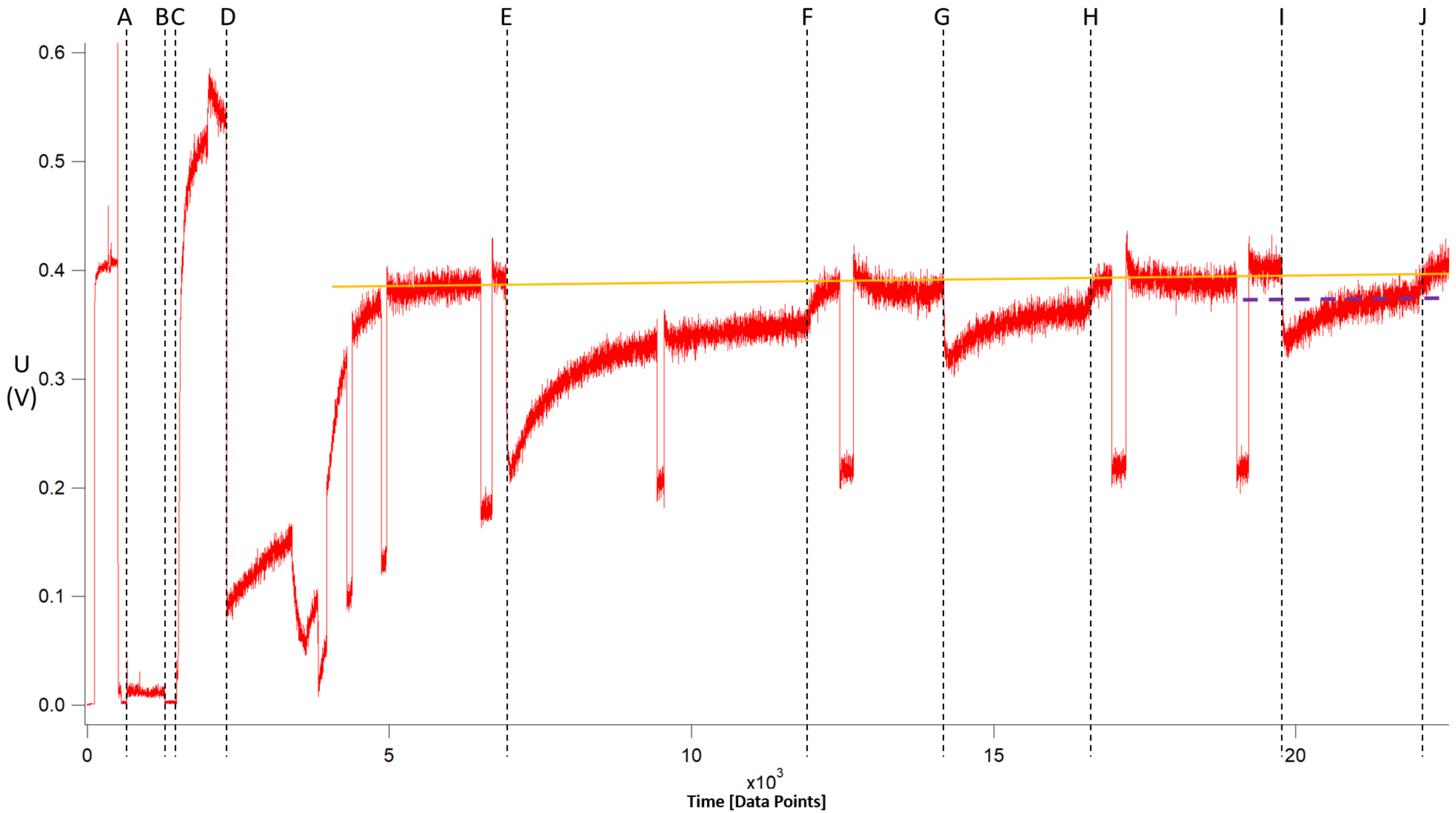

Figure S5. KFR Adsorption experiment of $PAXA_V$ and NH_2OH. X-axis (time) data points (4 Hz acquisition rate), y-axis Voltage. Yellow line: corresponding signal to steady-state-flow of gas molecules, purple dashed line: new "quasi"-steady-state. 0 - Turn on MS, A to B – background H_2O check at 14 mm, B to C – background at 1 mm, C – opening NH_2OH flow, D – Change to 1mm escape orifice and flow establishment + background checks (H_2O), E – opening of SC and 1st Uptake, F – Closed SC, F to G - Flow measurement, G to H – SC open and 2nd uptake, H to I – SC closed, flow measurement and H_2O checks, I to J – 3rd uptake and J – SC closed

Upon the last adsorption experiment and flow measurement the SC stays closed. The probe gas supply is turned off (Figure S6, label L), the diameter of the escape orifice was set from 1 to 4 to 8 to 14 mm (Figure S6, label M, N and O), which increases the pumping speed and consequently transport of probe gas molecules exiting the reactor chamber via the escape orifice (Figure S6, part L to P) in order to reach the baseline faster by using a faster pumping rate. The pumping continues until the MS signal vanishes at the 14 mm orifice diameter and the operator changes manually the escape orifice last before the desorption experiment can start in the 1 mm orifice KFR (Figure S6, point P). In case one can expect a slow desorption, a larger escape orifice may be chosen. Once the plunger is lifted and the SC opened, the desorption experiment starts as visualized in Figure S6, label Q at the 1 mm orifice. As the signal decreases, the diameter of the escape orifice is correspondingly increased to 4, 8, 14 mm, Figure S6, label R, S and T respectively in order to detect all adsorbed molecules that will desorb at ambient temperature at a rate observable in this experiment. Upon opening the SC a small or large increase in voltage may be detected, in case molecules are present, owing to molecular desorption. A slow decrease is a sign for slow release from the sample while a quick, strong signal highlights a strong, spontaneous desorption. The first change of the escape orifice causes generally a strong signal, due to the release of probe gas molecules, which escape into the MS-detection chamber across the orifice, while also interrupting the molecular beam for a few seconds, Figure S6, label R.

The post-experiment data processing includes the integration of adsorption and desorption experiments to calculate the total number of molecules that reacted with the substrate material, also in regards to permanent or short-term. The normalization of results to (# molecules cm^{-2}) is possible since the mass and exact BET surface area have been determined beforehand.

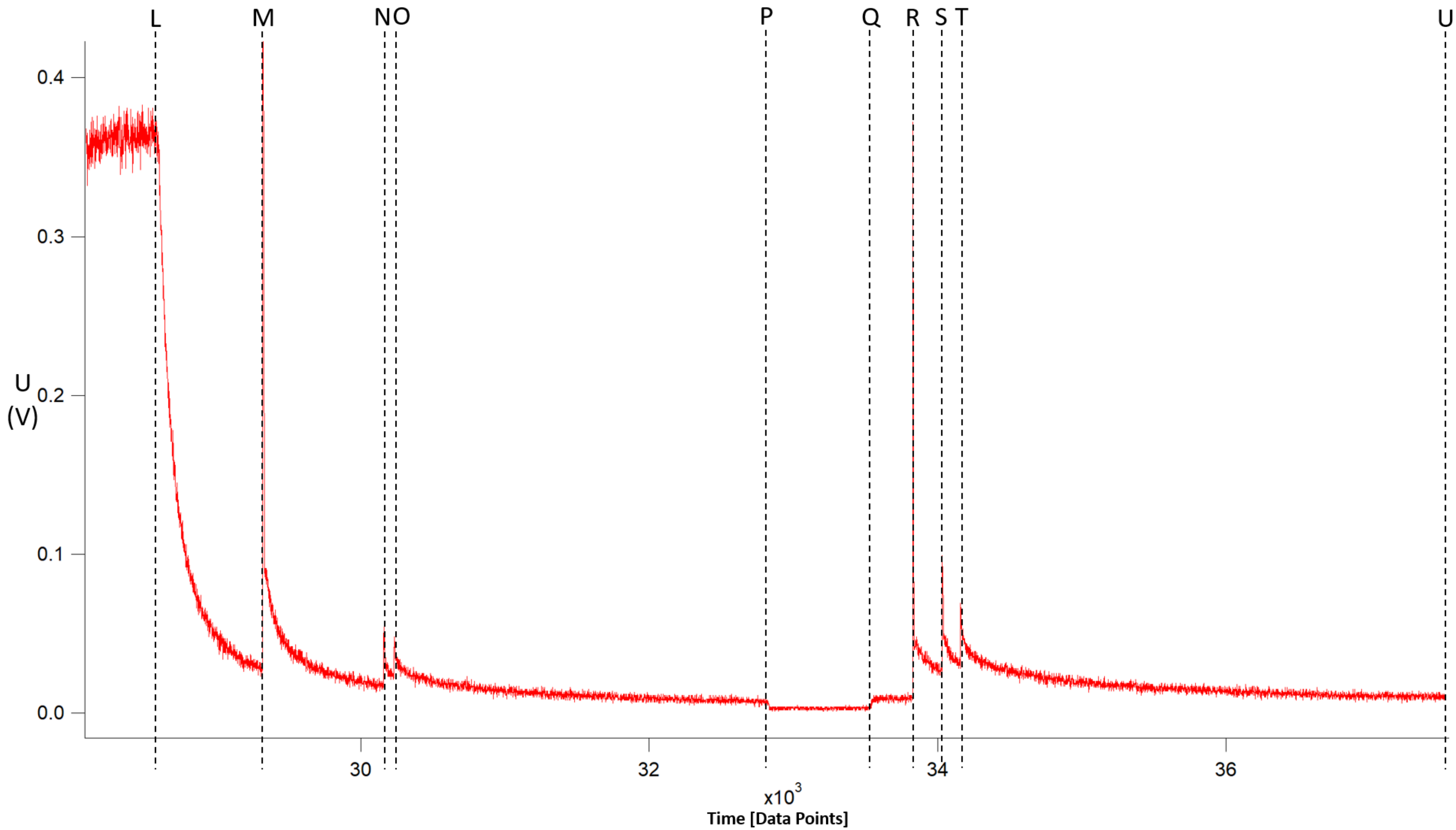

Figure S6. KFR Desorption experiment of $PAXA_{SS}$ and NH_2OH. X-axis data points (4 Hz acquisition rate), y-axis Voltage. L – Turn off NH_2OH flow, M – change escape orifice 1 to 4 mm, N – change escape orifice 4 to 8 mm, O – change escape orifice 8 to 14 mm, P – Change to 1 mm orifice, Q – opening of SC, R – Change to 4 mm orifice, S – change to 8 mm orifice, T – change to 14 mm escape orifice, U - end of experiment.

KFR - IGOR experimental curves

ESI figure S7 and S8 display the uptake curves of $PAXA_V$ and $PAXA_{UV}$ when being exposed to a steady flow of TFA-molecules. The solid yellow line indicates the initial flow of molecules, while voltage is directly proportional to number of molecules detected by the MS. At timepoint $20.31*10^3 \approx 85$ min (4 Hz acquisition frequency) the SC is manually opened by lifting the plunger and the drop in voltage is displaying the initial reactivity of TFA with $PAXA_V$. Once fast reacting SFGs are saturated the number of molecules detected by the MS, and therefore the signal steadily increases (k_{esc} increases) until the initial flow is reached again or a new "quasi"-steady-state (orange dashed line) has emerged; suggesting a relatively slow transport mechanism into the bulk of the material ($k_{het\text{-}StSt}$). At timepoint $25.3*10^3 \approx 105$ min the SC was closed. Consequently, the previous steady-state flow of molecules was met once again (100% k_{esc}).

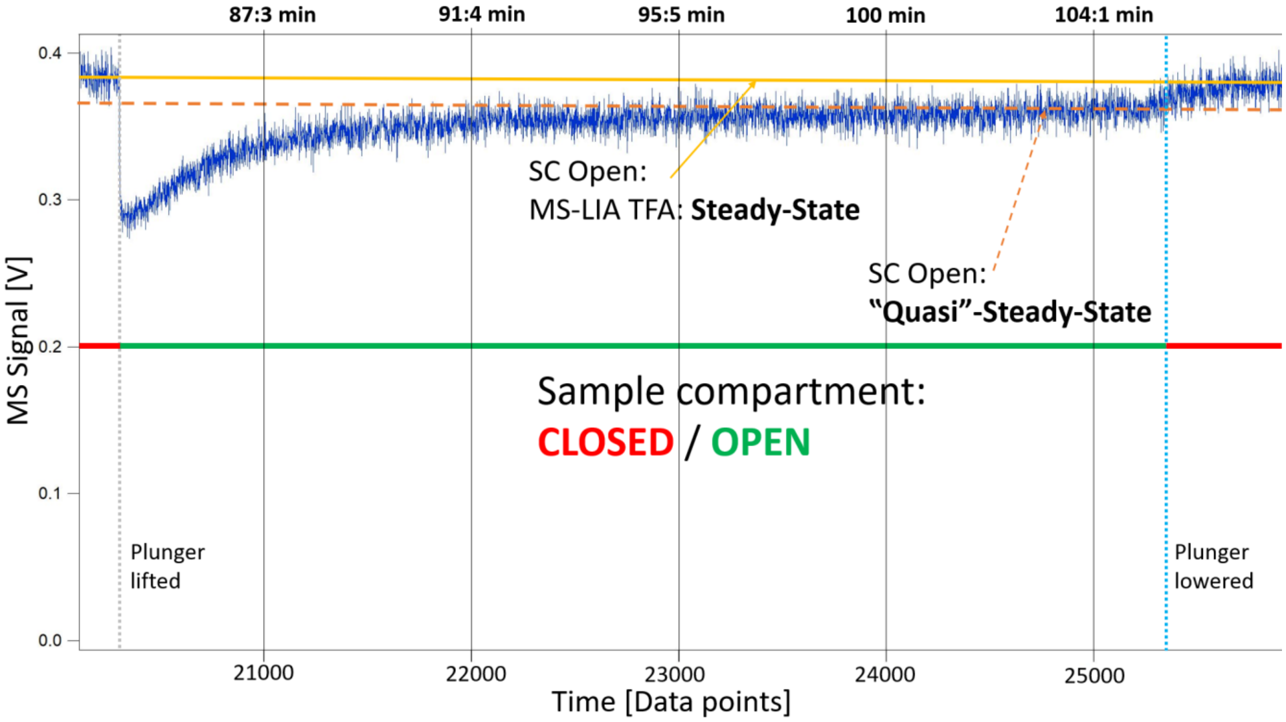

Figure S7. Knudsen Flow Reactor Uptake phase of $PAXA_V$ + TFA

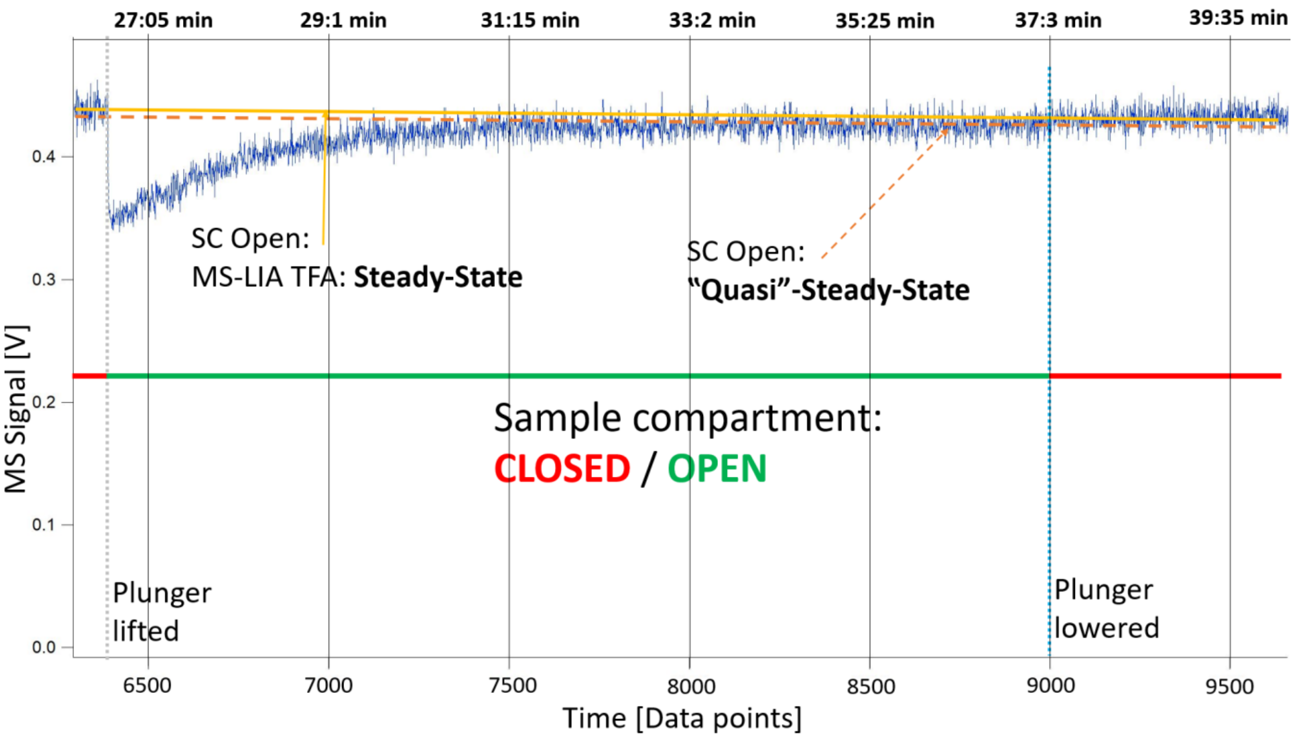

Figure S8. Knudsen Flow Reactor Uptake phase of $PAXA_{UV}$ + TFA

H_2O molecule affinity increased for both polymers after laboratory UV light exposure: $PAXA_V$ showed initially a higher surface reactivity with an uptake of $1.6*10^{13}$ molecule/cm^2 compared to $6.28*10^{12}$ molecule/cm^2 of PBX_V, displaying a factor of 2.55. UV aged $PAXA_{UV}$ reacted with $9.48*10^{13}$ molecule/cm^2, a 5.93-times increase to its virgin state while also developing a new quasi-steady-state, similarly PBX_{UV} reactivity increased 7.75 times to $4.87*10^{13}$ molecule/cm^2 likewise showing a new quasi-steady-state. **TMA** is a strong base and used to test for weak and strong acidic SFGs. $PAXA_V$ ($5.4*10^{12}$ molecule/cm^2) shows a 2.44times higher reactivity compared to PBX_V ($2.21*10^{12}$ molecule/cm^2), while $PAXA_{UV}$ ($1.28*10^{13}$ #molecule/cm^2) shows only a 1.44times higher reactivity in contrast to PBX_{UV} ($8.91*10^{12}$ #molecule/cm^2). **TFA** is a strong acid and used to test for weak and strong basic SFGs. $PAXA_V$ ($3.28*10^{13}$ #molecule/cm^2) shows a 13.56times higher initial reactivity than PBX_V ($2.42*10^{12}$ #molecule/cm^2) when tested for basic surface functionality. After UV exposure the factor between $PAXA_{UV}$ ($1.14*10^{13}$ #molecule/cm^2) and PBX_{UV} ($8.05*10^{12}$ #molecule/cm^2) decreased to only 1.42: due to a significant loss in reactivity of $PAXA_{UV}$ and increase in reactivity of PBX_{UV}, also highlighted by the significant "quasi"-steady-state of $PAXA_V$ and only marginal "quasi"-steady-state of $PAXA_{SS}$. **NO_2** molecule uptake is relatively low and changed in opposite directions upon weathering: $PAXA_{UV}$ ($2.43*10^{12}$ #molecule/cm^2) shows no longer a "quasi"-steady-state and reacts with only 0.79-fold the initial amount of $PAXA_V$ ($1.22*10^{13}$ #molecule/cm^2). PBX_V ($1.05*10^{12}$ #molecule/cm^2) reactivity increases by 1.38-fold upon UV exposure: PBX_{UV} ($1.46*10^{12}$ #molecule/cm^2), without showing any bulk uptake. In both degradation states does PAXA show a higher reactivity. Selected repetitions of analysis showed an error of 5-10% in uptake.

HA: PBX_V shows a total adsorption of $7.45*10^{13}$ molecule/cm^2 and a small "quasi"-steady-state behavior; The uptake is 1.08-fold increased, to $8.09*10^{13}$ molecule/cm^2 for PBX_{UV}, which also shows a significant "quasi"-steady-state behavior, underlining a faster bulk-transport phenomenon compared to the virgin state. $PAXA_V$ displays an initial adsorption of $3.28*10^{14}$ molecule/cm^2 and also a slight "quasi"-steady-state, whereas $PAXA_{UV}$ displays a more significant "quasi"-steady-state and uptake with $8.09*10^{13}$ molecule/cm^2, which is four times less than $PAXA_V$. $PAXA_V$ shows a 4.4 times higher HA molecule reactivity than PBX_V, whereas $PAXA_{UV}$ and PBX_{UV} show identical HA reactivities. The increases in "quasi"-steady-state behavior lead to the change of the characteristic rate constants $k_{het\text{-}StSt}$ and $\gamma_{BET\text{-}StSt}$ after UV degradation, here the change is more

significant for PBX. Table S1 shows the low reactivity for both polymers when exposed to **O_3**. $PAXA_V$ (1.22*10^{13} molecule/cm^2) has a 2.33-times higher initial reactivity than PBX_V (5.22*10^{12} molecule/cm^2). PBX_{UV} takes up 1.15*10^{13} molecule/cm^2, a 2.2-times increase relative to PBX_V. $PAXA_{UV}$ (2.28*10^{13} molecule/cm^2) reactivity towards O_3 as a probe gas is 1.87-fold increased to its virgin state and increases 1.98-fold relative to PBX_{UV}. Both virgin polymers show a "quasi"-steady-state uptake of O_3 molecules, resulting in $k_{het\text{-}StSt}$ and $\gamma_{BET\text{-}StSt.}$ The total coverage of probe gas specific SFGs is calculated dividing the number of adsorbed molecules by the value of one molecular monolayer at saturation using values reported by Mirghaffari et al., 2021. Table S1 displays artificial UV degraded polymers **with and without O_3 pre-exposure**. PBX_{UV} and $PAXA_{UV}$ show a similar uptake of 8.09*10^{13} molecule/cm^2; after O_3 degradation the molecular uptake increased for both cases by 5%. Both polymers and all four degradation-states show a "quasi"-steady-state uptake and transport into the bulk material.

PBX_V shows an adsorption of 7.45*10^{13} #molecule/cm^2 and a slight "quasi"-steady-state uptake (small value of $k_{het\text{-}StSt}$ and $\gamma_{BET\text{-}StSt}$). The uptake is increased by 1.02-times to 7.6*10^{13} #molecule/cm^2 for PBX_{V+O3}, which is within the error tolerance of ±7%, but shows a distinct "quasi"-steady-state uptake. $PAXA_V$ displays an adsorption of 3.28*10^{14} #molecule/cm^2 and also a slight "quasi"-steady-state. $PAXA_{V+O3}$ shows a similar "quasi"-steady-state uptake with only 6.82*10^{13} #molecule/cm^2, which is approximately 5-times less than for pre-degradation conditions. $PAXA_V$ shows 4.4-times more HA-reactive SFGs than PBX_V, whereas $PAXA_{V+O3}$ shows a slightly lower reactivity with only 90% of PBX_{V+O3}-value. Values for $k_{het\text{-}StSt}$ and $\gamma_{BET\text{-}StSt}$ show only little change in the case of PAXA, suggesting a significant loss in fast-reacting i-OH groups upon UV exposure on the surface.

H_2O	PBX_V	PBX_{UV}	$PAXA_V$	$PAXA_{UV}$
**Uptake** (# of molecule/cm^2)	6.28E+12	4.87E+13	1.60E+13	9.48E+13
**Increase/Decrease** w/r virgin polymer	1	7.75	1	5.93
Factor between **virgin** polymers	2.55			
Factor between **degraded** polymers		1.95		
**"Quasi"**-Steady State	No	Yes, min.	No	Yes, min.
$k_{heterogeneous\text{-}Steady\text{-}State}$ / s^{-1}		7.80E-04		1.54E-03
$\gamma_{BET\text{-}Steady\text{-}State}$		3.19E-08		8.43E-08

TMA	PBX_V	PBX_{UV}	$PAXA_V$	$PAXA_{UV}$
**Uptake** (# of molecule/cm^2)	2.21E+12	8.91E+12	5.39E+12	1.28E+13
**Increase/Decrease** w/r virgin polymer	1	4.04	1	2.38
Factor between **virgin** polymers	2.44			
Factor between **degraded** polymers		1.44		
**"Quasi"**-Steady State	No	No	No	Yes, min.
$k_{heterogeneous\text{-}Steady\text{-}State}$ / s^{-1}				4.02E-04
$\gamma_{BET\text{-}Steady\text{-}State}$				3.99E-08

TFA	PBX_V	PBX_{UV}	$PAXA_V$	$PAXA_{UV}$
**Uptake** (# of molecule/cm^2)	2.42E+12	8.05E+12	3.28E+13	1.14E+13
**Increase/Decrease** w/r virgin polymer	1	3.33	1	0.35
Factor between **virgin** polymers	13.56			
Factor between **degraded** polymers		1.42		
"**Quasi**"-Steady State	No	No	Yes	Yes, min.
$k_{heterogeneous-Steady-State}$ / s^{-1}			2.07E-04	5.13E-04
$\gamma_{BET-Steady-State}$			1.13E-07	7.06E-08

HA	PBX_V	PBX_{UV}	$PAXA_V$	$PAXA_{UV}$
**Uptake** (# of molecule/cm^2)	7.45E+13	8.09E+13	3.28E+14	8.09E+13
**Increase/Decrease** w/r virgin polymer	1	1.08	1	0.25
Factor between **virgin** polymers	4.40			
Factor between **degraded** polymers		1.00		
"**Quasi**"-Steady State	Yes, min.	Yes	Yes, min.	Yes
$k_{heterogeneous-Steady-State}$ / s^{-1}	7.43E-04	6.81E-03	1.82E-03	3.12E-03
$\gamma_{BET-Steady-State}$	1.09E-08	3.76E-07	5.35E-08	2.31E-07

NO_2	PBX_V	PBX_{UV}	$PAXA_V$	$PAXA_{UV}$
**Uptake** (# of molecule/cm^2)	1.050E+12	1.457E+12	3.065E+12	2.425E+12
**Increase/Decrease** w/r virgin polymer	1	1.39	1	0.79
Factor between **virgin** polymers	2.92			
Factor between **degraded** polymers		1.66		
"**Quasi**"-Steady State	No	No	Yes	No
$k_{heterogeneous-Steady-State}$ / s^{-1}			1.99E-04	
$\gamma_{BET-Steady-State}$			3.44E-09	

O_3	PBX_V	PBX_{UV}	$PAXA_V$	$PAXA_{UV}$
**Uptake** (# of molecule/cm^2)	5.22E+12	1.15E+13	1.22E+13	2.28E+13
**Increase/Decrease** w/r virgin polymer	1	2.20	1	1.87
Factor between **virgin** polymers	2.33			
Factor between **degraded** polymers		1.98		
"**Quasi**"-Steady State	Yes	No	Yes	No
$k_{heterogeneous-Steady-State}$ / s^{-1}	6.11E-04		9.17E-04	
$\gamma_{BET-Steady-State}$	8.92E-09		2.69E-08	

HA & Virgin Polymer + Short-term O_3 degradation	PBX_V	PBX_{V+O3}	$PAXA_V$	$PAXA_{V+O3}$
**Uptake** (# of molecule/cm^2)	7.45E+13	7.60E+13	3.28E+14	6.82E+13
**Increase/Decrease** w/r virgin polymer	1	1.02	1	0.21
Factor between **virgin** polymers	4.40			
Factor between **degraded** polymers		0.90		
"**Quasi**"-Steady State	Yes, min.	Yes	Yes, min.	Yes
$k_{heterogeneous\text{-}Steady\text{-}State}$ / s^{-1}	7.43E-04	2.14E-03	9.17E-04	1.28E-03
$\gamma_{BET\text{-}Steady\text{-}State}$	1.09E-08	3.13E-08	5.35E-08	3.77E-08

HA & UV-degraded Polymer + Short-term O_3 degradation	PBX_{UV}	PBX_{UV+O3}	$PAXA_{UV}$	$PAXA_{UV+O3}$
**Uptake** (# of molecule/cm^2)	8.09E+13	8.49E+13	8.09E+13	8.49E+13
**Increase/Decrease** w/r virgin polymer	1	1.05	1	1.05
Factor between **virgin** polymers	1.00			
Factor between **degraded** polymers		1.00		
"**Quasi**"-Steady State	Yes	Yes	Yes	Yes
$k_{heterogeneous\text{-}Steady\text{-}State}$ / s^{-1}	6.81E-03	6.51E-03	3.12E-03	2.30E-03
$\gamma_{BET\text{-}Steady\text{-}State}$	3.76E-07	3.60E-07	2.31E-07	1.71E-07

Table S1. Full list of reactivities

	PBX_V		PBX_{UV}		$PAXA_V$		$PAXA_{UV}$	
	$k_{het\text{-}StSt}$	$\gamma_{BET\text{-}StSt}$	$k_{het\text{-}StSt}$	$\gamma_{BET\text{-}StSt}$	$k_{het\text{-}StSt}$	$\gamma_{BET\text{-}StSt}$	$k_{het\text{-}StSt}$	$\gamma_{BET\text{-}StSt}$
H_2O	-	-	7.80E-04	3.19E-08	-	-	1.54E-03	8.43E-08
TMA	-	-	-	-	-	-	4.02E-04	3.99E-08
TFA	-	-	-	-	2.07E-04	1.13E-07	5.13E-04	7.06E-08
HA	7.43E-04	1.09E-08	6.81E-03	3.76E-07	1.82E-03	5.35E-08	3.12E-03	2.31E-07
NO_2	-	-	-	-	1.99E-04	3.44E-09	-	-
O_3	6.11E-04	8.92E-09	-	-	9.17E-04	2.69E-08	-	-

Table S2. Individual rate coefficient of uptake ($k_{het\text{-}StSt}$ in s^{-1}) and uptake probability of SSA ($\gamma_{BET\text{-}StSt}$).

ATR-FTIR results

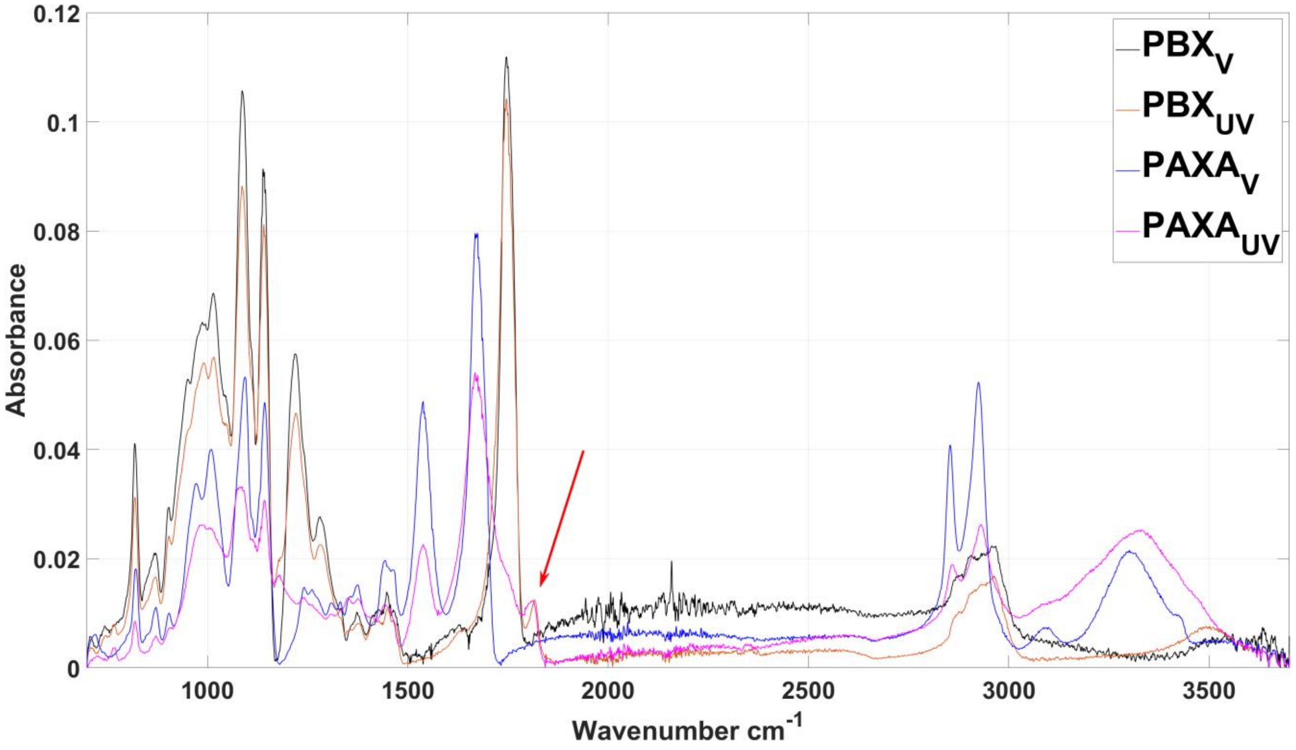

Figure S9. ATR-FTIR spectra of PBX$_V$, PBX$_{UV}$, PAXA$_V$ and PAXA$_{UV}$

The spectral fingerprint region between 700-1500cm^{-1} discloses similar peak patterns for PBX$_V$ and PAXA$_V$, most plausibly due to the shared DGAX-molecule. With increasing wavenumber intrinsic peaks vary for each polymer. UV light has an effect on both polymers by decreasing the overall signal intensity and creating several new peaks: both polymers show one distinct shared new peak around 1814cm^{-1} (C=O). PBX$_V$ shows distinct peaks at 819cm^{-1} (C=C), 1016.5cm^{-1} (C=C), 1088cm^{-1} (C-O), 1042cm^{-1} (C-O), 1223cm^{-1} (C-O), 1748cm^{-1} (C=O) and a broad one at 2969cm^{-1} (C-H). PAXA$_V$ shares peaks at 831cm^{-1} (C=C), 1014cm^{-1} (C=C), 1096cm^{-1} (C-O), 1141.5cm^{-1} (C-O) with PBX$_V$ and shows unique ones at 1539cm^{-1} (N-O), 1674.5cm^{-1} (C=O), 2854.5cm^{-1} (C-H), 2925cm^{-1} (C-H), 3095cm^{-1} (C-H) and abroad one 3303cm^{-1} (N-H).

Spectra of PBX$_{UV}$ display most of the specific fingerprint peaks, but also an overall loss in intensity and sharpness as displayed in ESI figure S10. Except for a more intense C-O stretching around wave number 1180cm^{-1} and a sharp new peak at 1817cm^{-1} (C=O) and a broad bump at 3500cm^{-1} (O-H).

PAXA$_{UV}$ also experienced a loss in spectral intensity and sharpness, but the peak pattern is more distinct after experiencing 25 days of artificial sunlight exposure. New peaks appear around 1814cm^{-1} (C=O) and a broad band from 3030-3500cm^{-1} (O-H) with peak at 3350cm^{-1} (N-H), displayed in ESI figure S11.

In the case of short-term Ozone treated PAXA and PBX in virgin and UV treated state no new absorbance bands could be identified, ATR-FTIR spectra are displayed in ESI figure S12 and S13, respectively. The authors want to **note** differences in intensity of ATR-FTIR absorbance were experienced throughout this study: pellet packing below the stamp above the ATR crystal are most likely the reason, especially for UV treated samples.

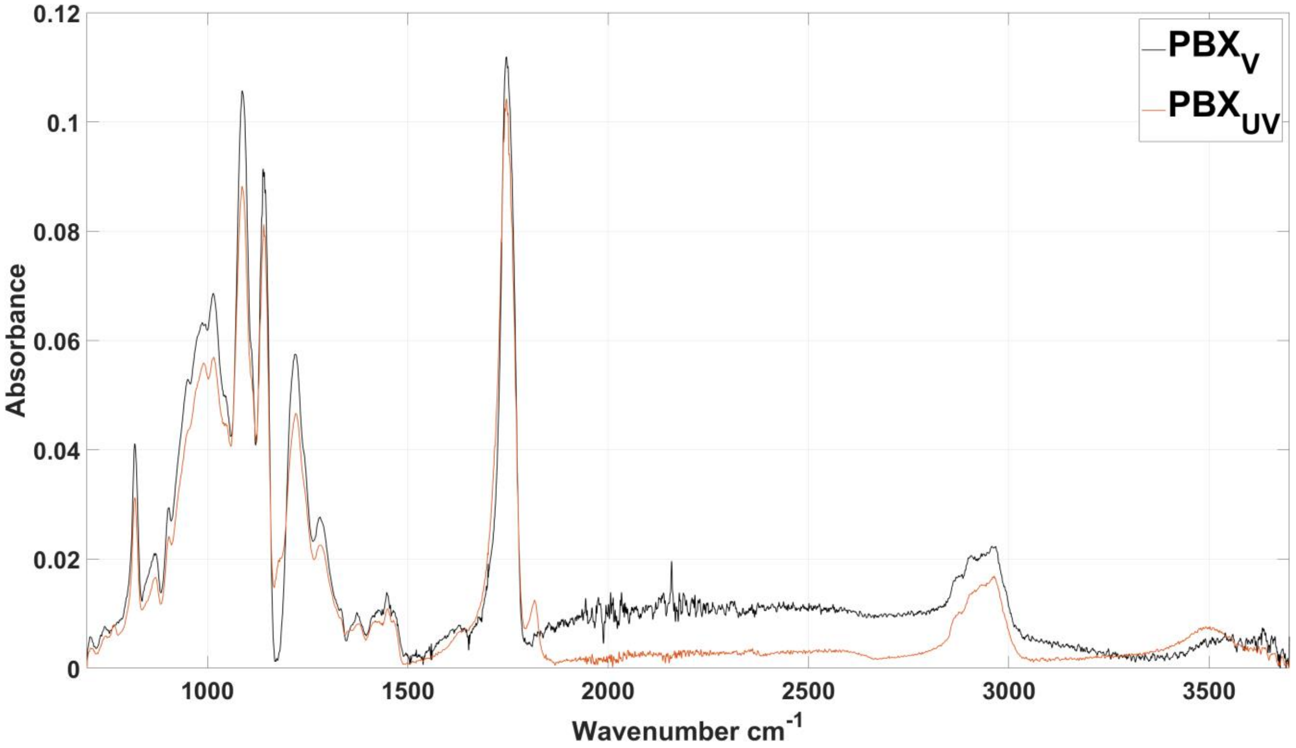

Figure S10. FT-IR ATR Spectra of PBX$_V$ & PBX$_{UV}$

PBX$_{UV}$ displays most of the specific fingerprint peaks, but also an overall loss in intensity and sharpness. Except for a more intense C-O stretching around wave number 1180cm^{-1} and a sharp new peak at 1817cm^{-1} (C=O) and a broad bump at 3500cm^{-1} (O-H).

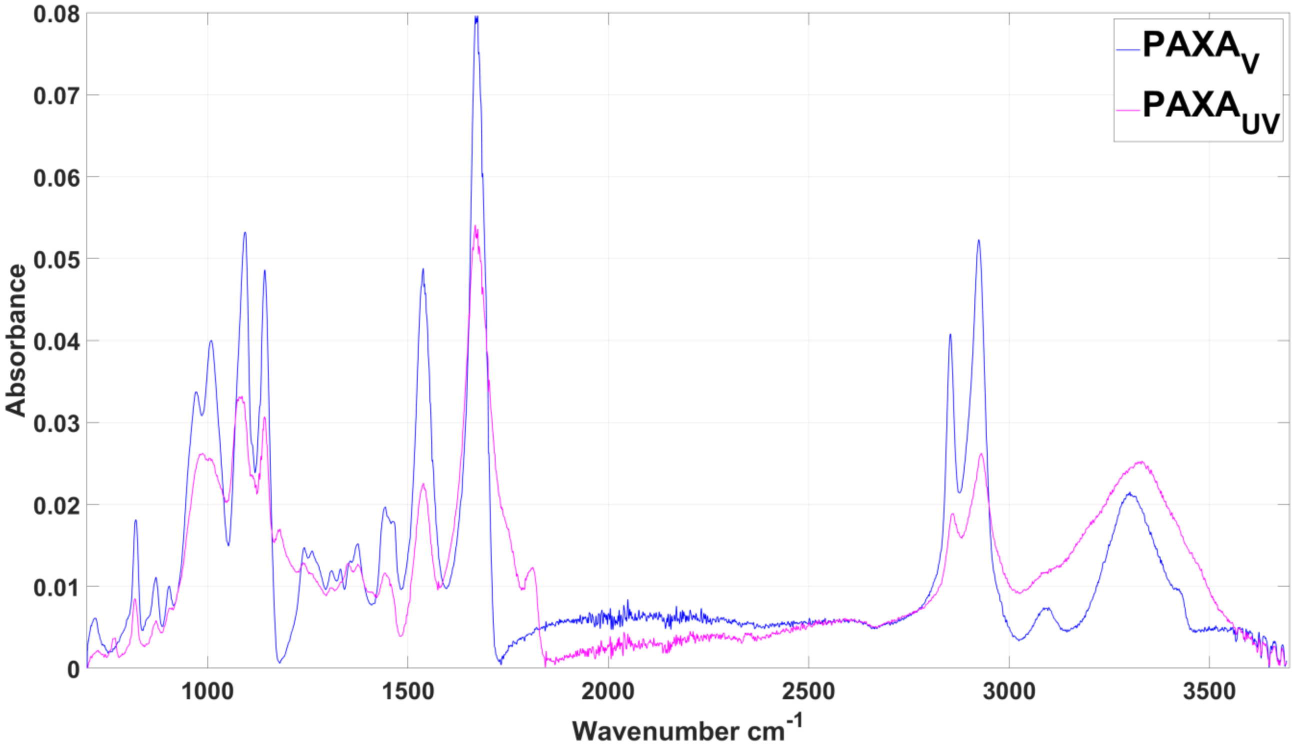

Figure S11. FT-IR ATR Spectra of PAXA$_V$ vs. PAXA$_{UV}$

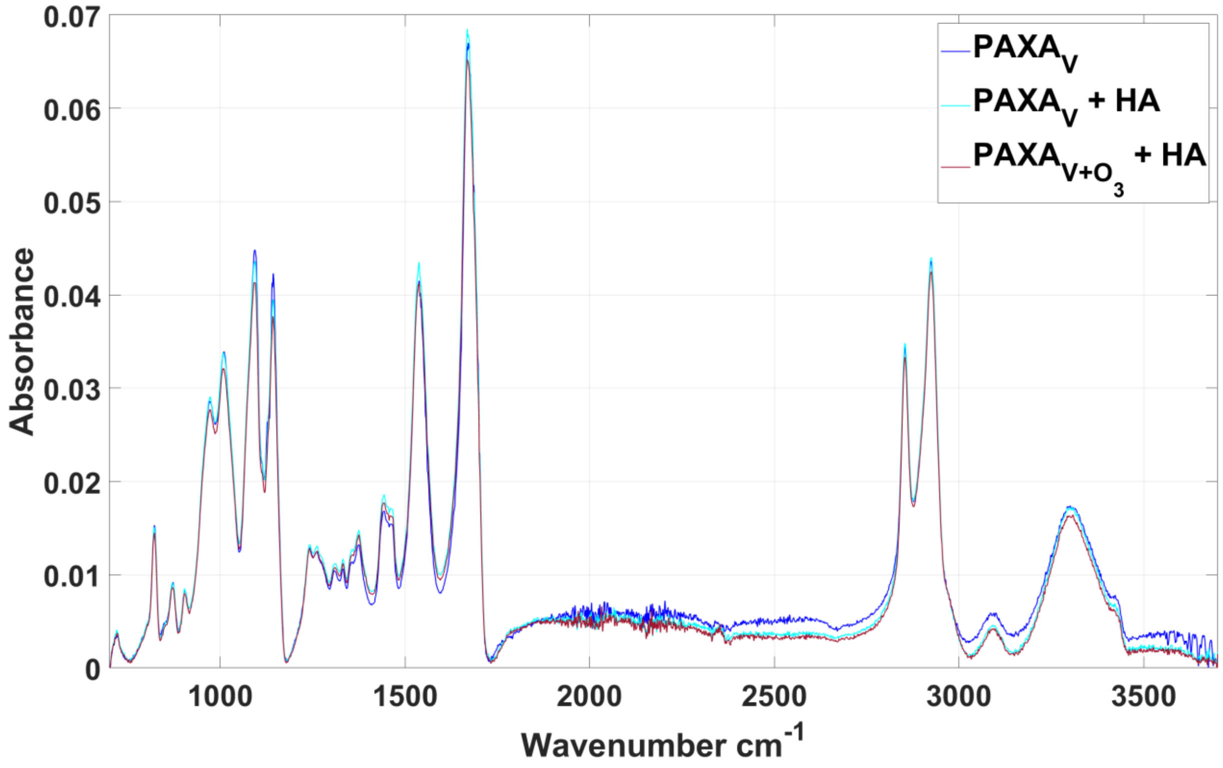

Figure S12. ATR-FTIR Spectra of $PAXA_V$, $PAXA_V$ after HA exposure ($PAXA_V$ + HA) and $PAXA_V$ pre-degraded by O_3, followed by HA exposure ($PAXA_{V+O3}$ + HA).

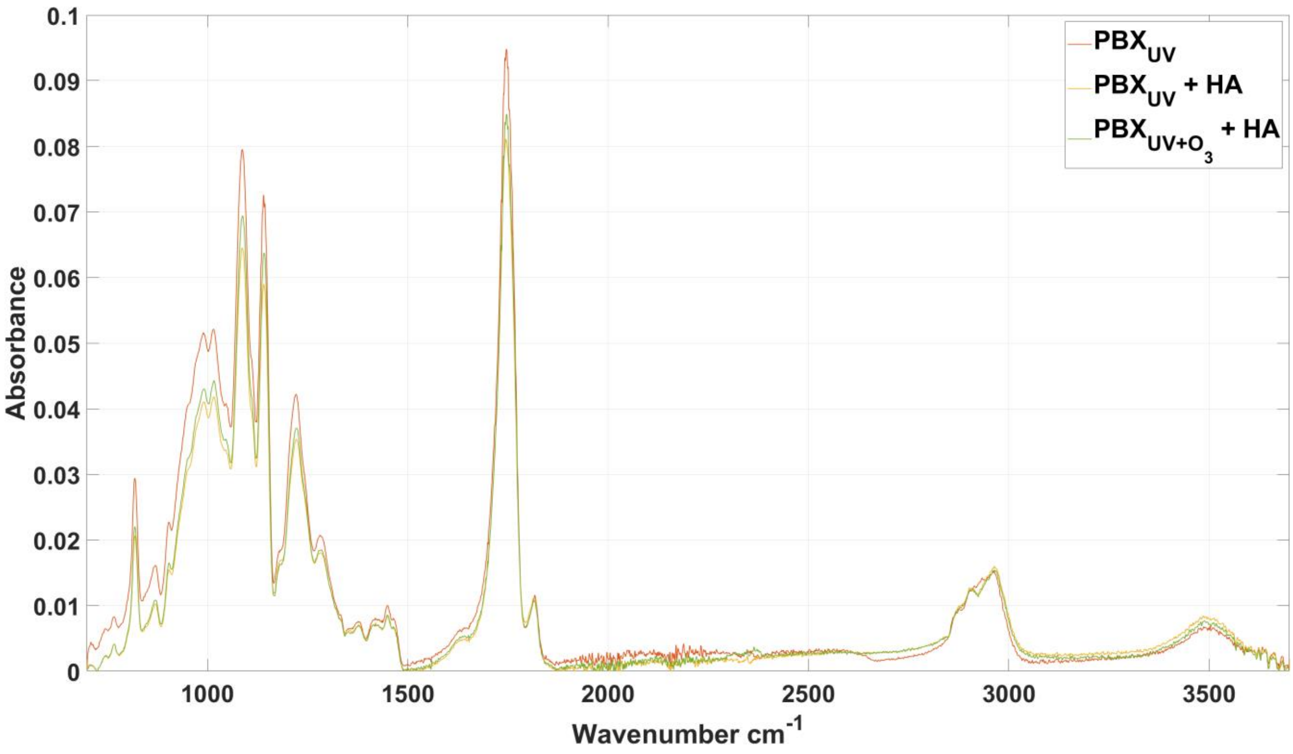

Figure S13. ATR-FTIR Spectra of PBX_{UV}, PBX_{UV} after HA exposure ($PAXA_V$ + HA) and PBX_{UV} pre-degraded by O_3, followed by HA exposure (PBX_{UV+O3} + HA).

PSA

PSA experiments for PBX reveal a slight shift towards smaller mean particle sizes: 0.821µm after being treated with UV light, while 1.017µm for PBX_V. Due to material properties and particle coagulation the analysis of PAXA was not possible using the identical protocol. Regarding BET and SEM results, the authors tend to think there are similarities in material behavior for PBX and PAXA; a shift towards larger mean particle sizes.

	**Mean (µm)**	**Median (µm)**
PBX_V	1.017	0.680
PBX_{UV}	0.821	0.624
$PAXA_V$	-	-
$PAXA_{UV}$	-	-

Table S3. Number Statistic (Arithmetic) values of particle size analysis. PAXA was not measurable due to particle coagulation.

SEM

SEM pictures visualize a decrease in details, mainly due to the decrease in total number of smaller particles and fine structures after laboratory sunlight exposure, as depicted in ESI Figure S14.

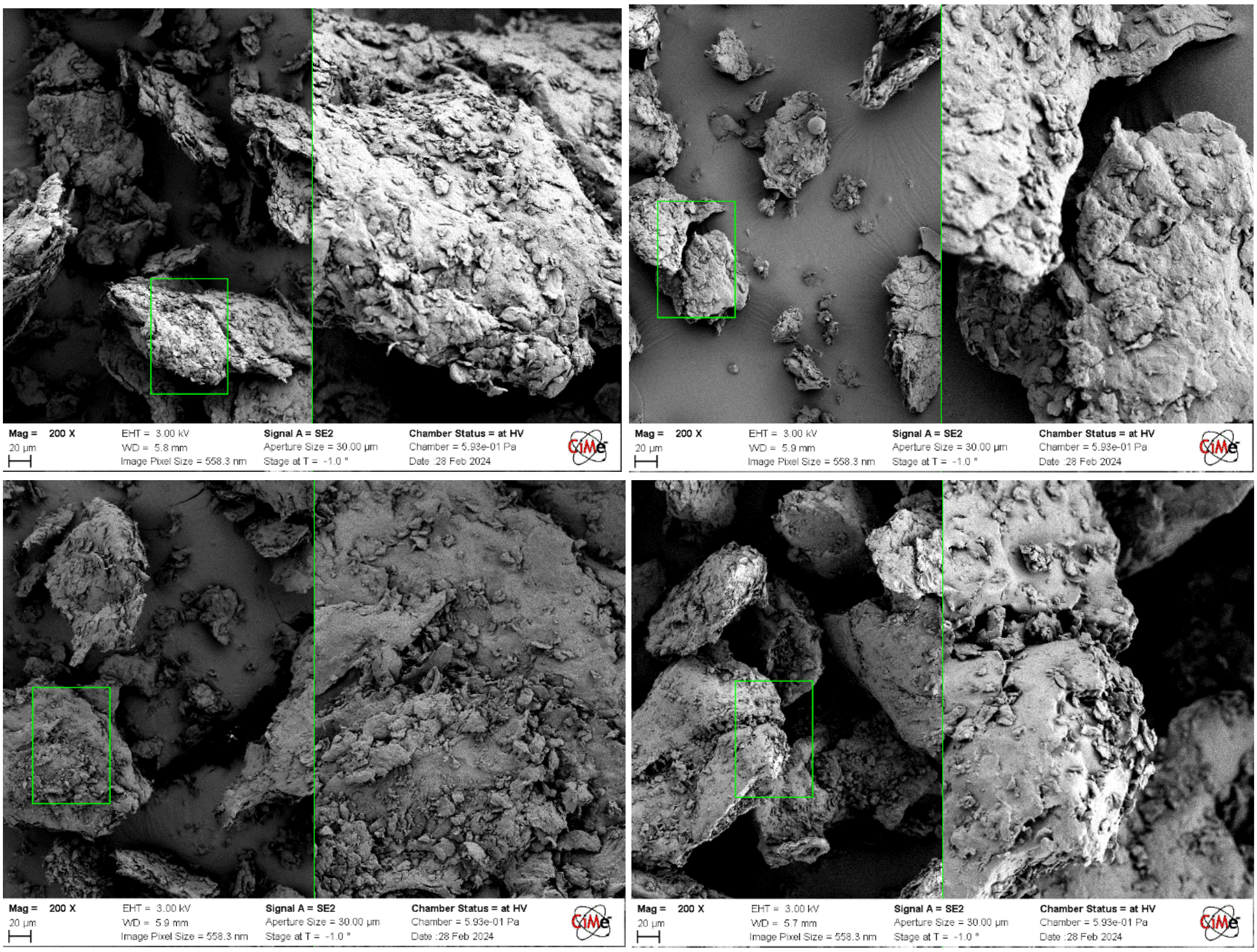

Figure S14. Top left PBX_V, top right PBX_{UV}, bottom left $PAXA_V$, bottom right $PAXA_{UV}$. PBX_V and $PAXA_V$ samples show similar particle morphology with fine and detailed structures atop of larger particles of varying sizes and shapes. Large particles appear to have rather sharp edges before and rounded, smoothed edges after UV treatment, which is more pronounced for PBX and might indicate the melting. Additionally, the overall

number of fine particles and structures atop larger ones decreases with UV light exposure, a general smoothing can be observed.

UV-Vis Diffuse Reflectance

UV-Vis was used to confirm and semi-quantify the visual impression of change in color for PBX and PAXA before/after laboratory UV exposure.

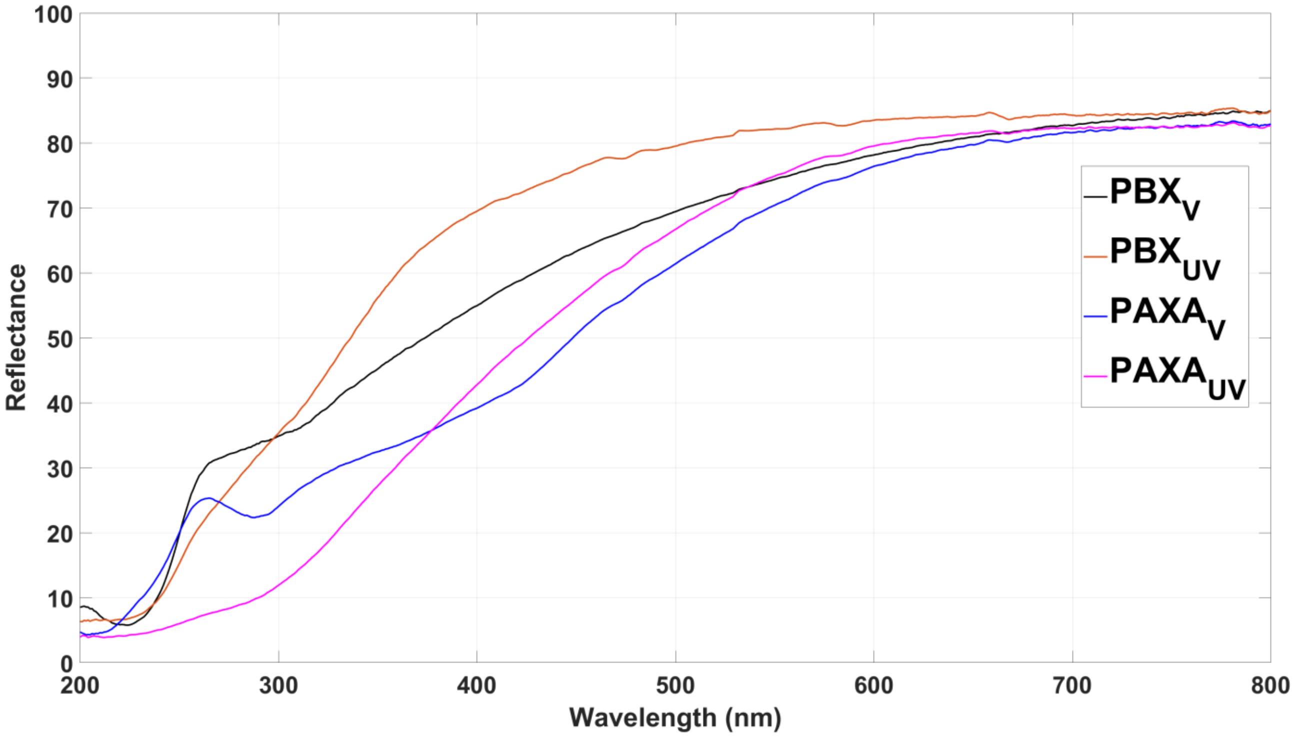

Figure S15. UV-Vis Diffuse Reflectance of polymers before/after aging

UV-Vis analysis has been used to 'semi-quantitatively' assess the change in color before/after ageing when degrading plastics with a laboratory UV source. PBX$_V$ and PAXA$_V$ show similar reflectance curve characteristics, with one closely resembling inflection point around 260nm. The PBX$_V$ reflectance shows overall less characteristics compared to PAXA$_V$, which also inherits inflection points around 430nm and 270nm. Inflection points at wavelengths 320nm and 260nm which are more pronounced in PBX$_V$ are also visible for PBX$_{UV}$ but weaker. For PBX$_{UV}$ the decrease in reflectance starts at lower wavelengths but the decline is more rapid. PAXA$_V$ and PAXA$_{UV}$ show a similar trend but curves are much closer, until 420nm. PAXA$_V$ specific inflection points ≈420nm, 320nm, 290nm and 260nm are smoothed out. PAXA$_{UV}$ shows the form of a constant descending slope: steep until 300nm, followed by a flattened area.

SEC-MALS

Material	Concentration (g/L)	*Mn* (kg/mol)	*Mp* (kg/mol)	*Mw* (kg/mol)	Polydispersity (*Mw* / *Mn*)	dn/dc (mL/g)
PBS_V	4.629	17.7	25.3	32.1	1.812	0.169
PBS_{UV}	5.601	2.4	1.5	5.4	2.247	0.169
$PAXA_V$	4.393	24.9	53.2	65.1	2.619	0.21
$PAXA_{UV}$	4.776	3.3	2.9	10.4	3.16	0.21

Table S4. SEC-MALS results: *Mn* - number average MW, *Mw* - weight average MW, *Mp* – MW of the highest peak. Data show degradation of MW performance indicators and broadening of chain length distribution upon UV degradation.

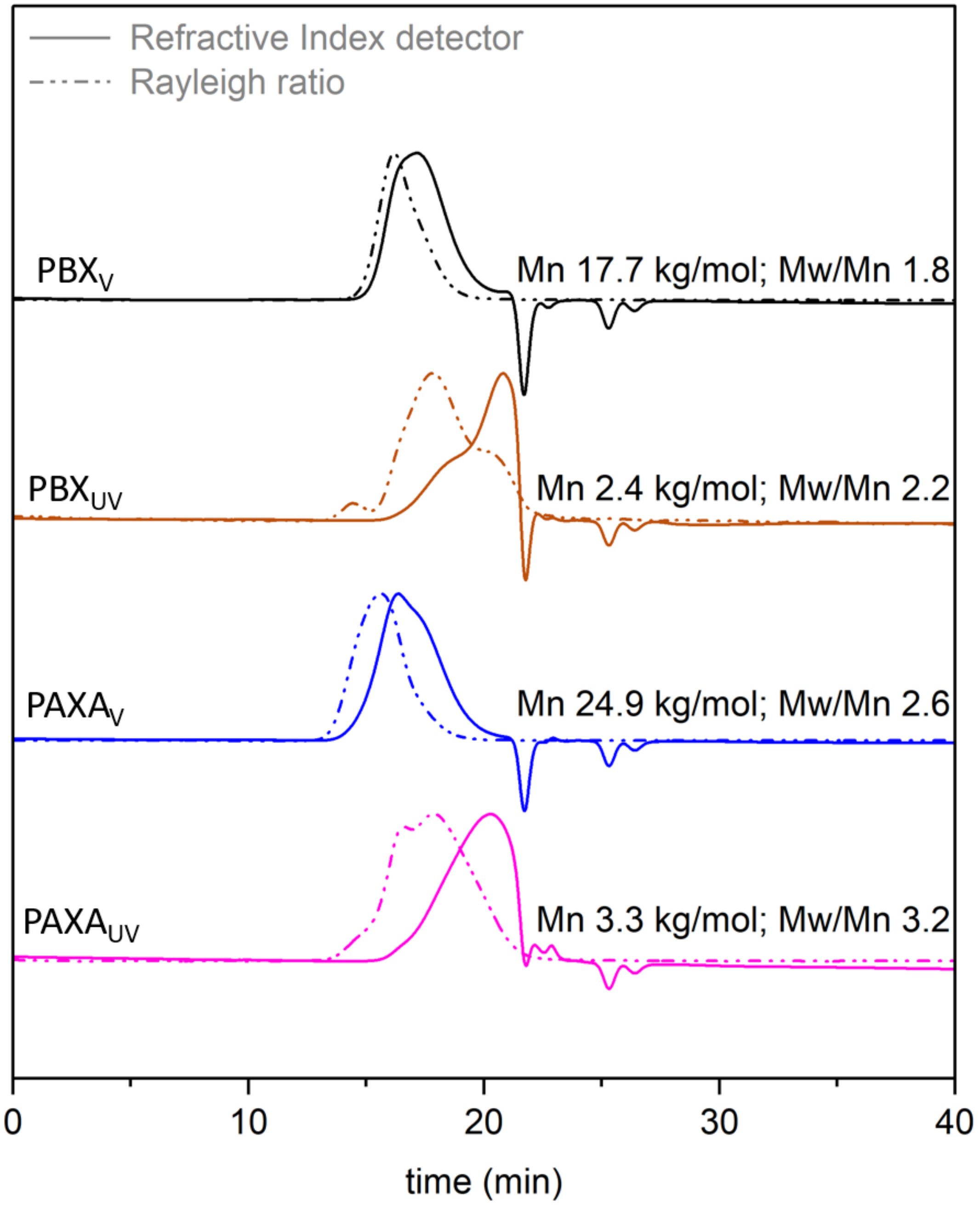

Figure S16. SEC-MALS chromatograms of PBX_V, PBX_{UV}, $PAXA_V$ and $PAXA_{UV}$.

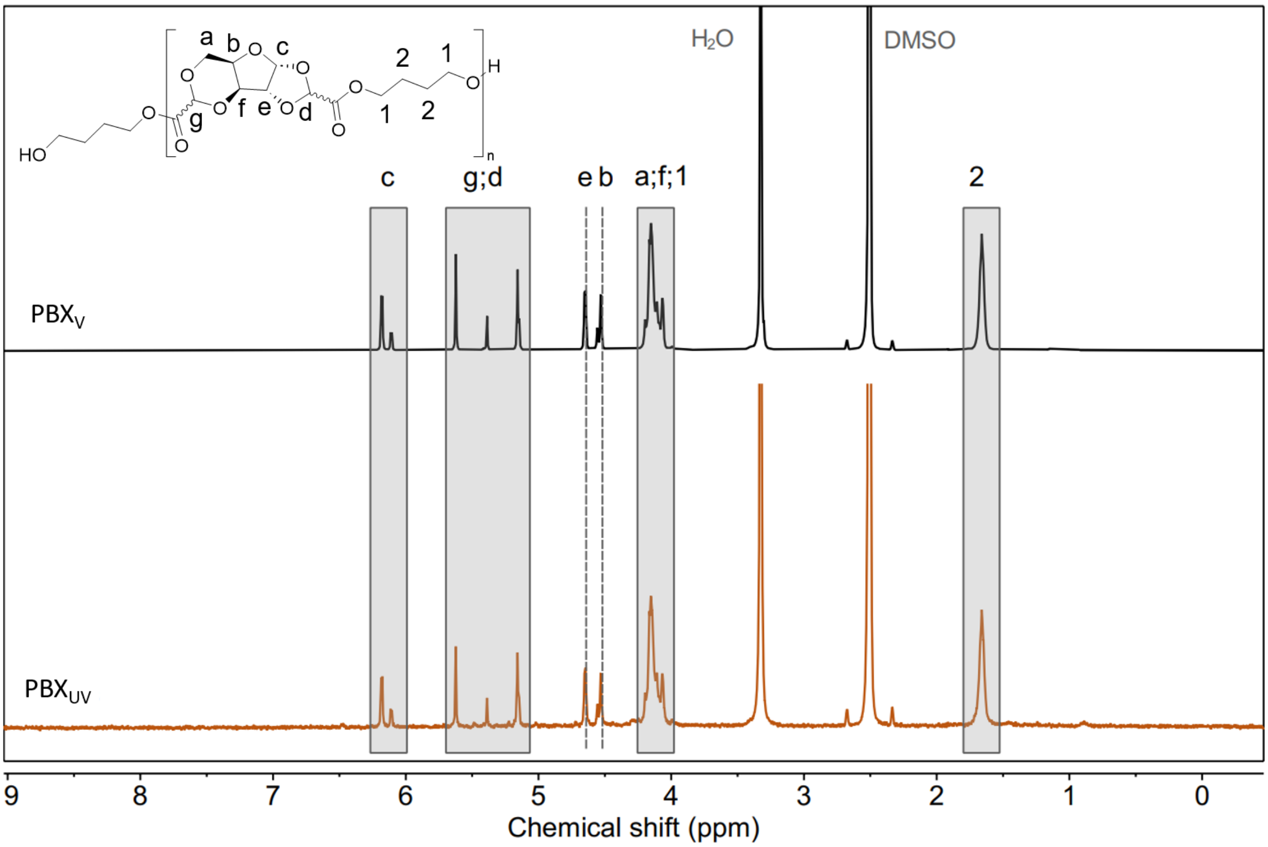

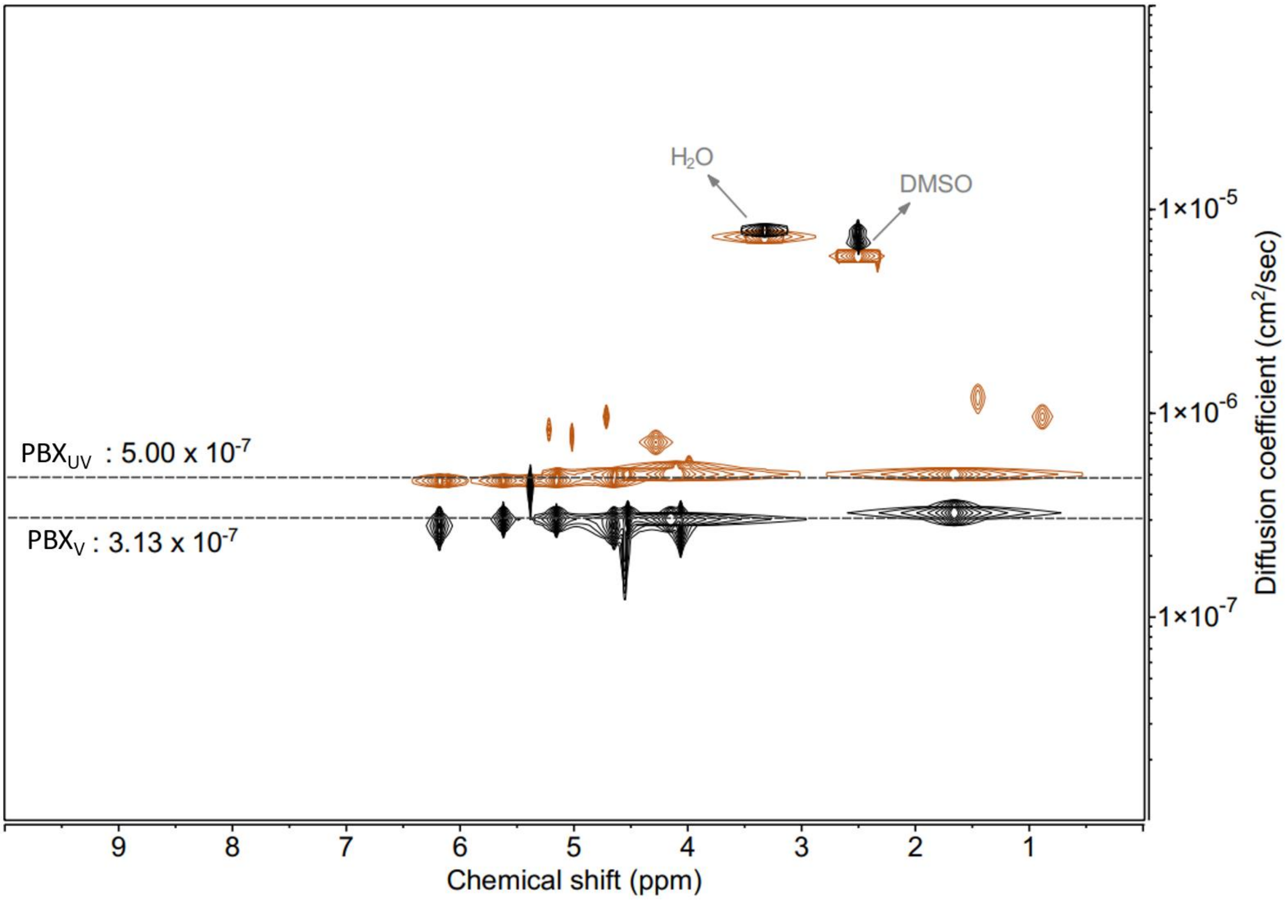

Figure S17. PBX_V and PBX_{UV}: ^{1}H-NMR (**top**) and 2D-DOSY-NMR (**bottom**): NMR experiments performed in d_6-dimethylsulfoxyde solvent: The black dotted lines represent the mean diffusion rate of the proton signals associated with the polymer backbone

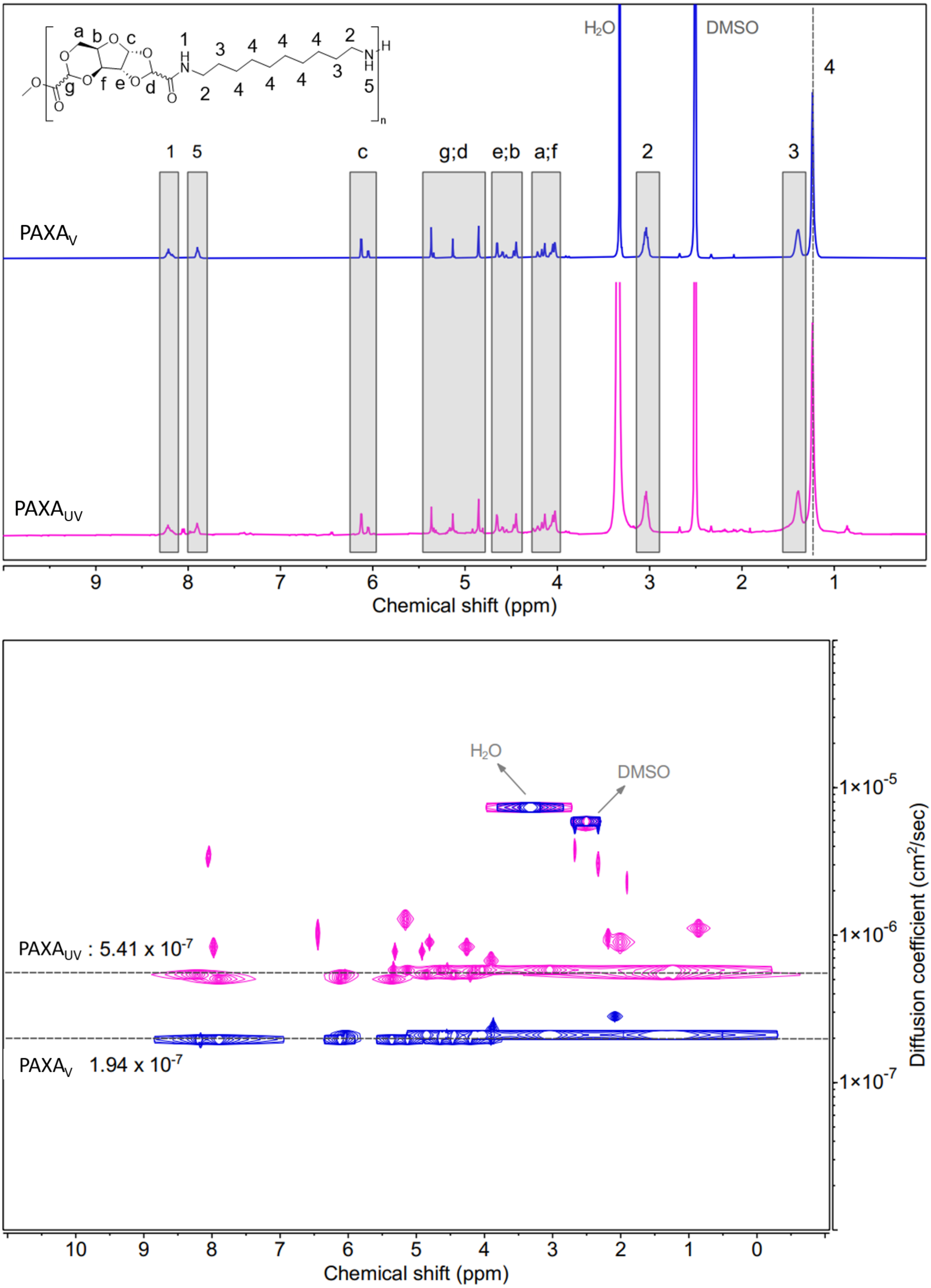

Figure S18. PAXA$_V$ and PAXA$_{UV}$: ^{1}H-NMR (**top**) and 2D-DOSY-NMR (**bottom**): NMR experiments performed in d$_6$-dimethylsulfoxyde solvent: The black dotted lines represent the mean diffusion rate of the proton signals associated with the polymer backbone

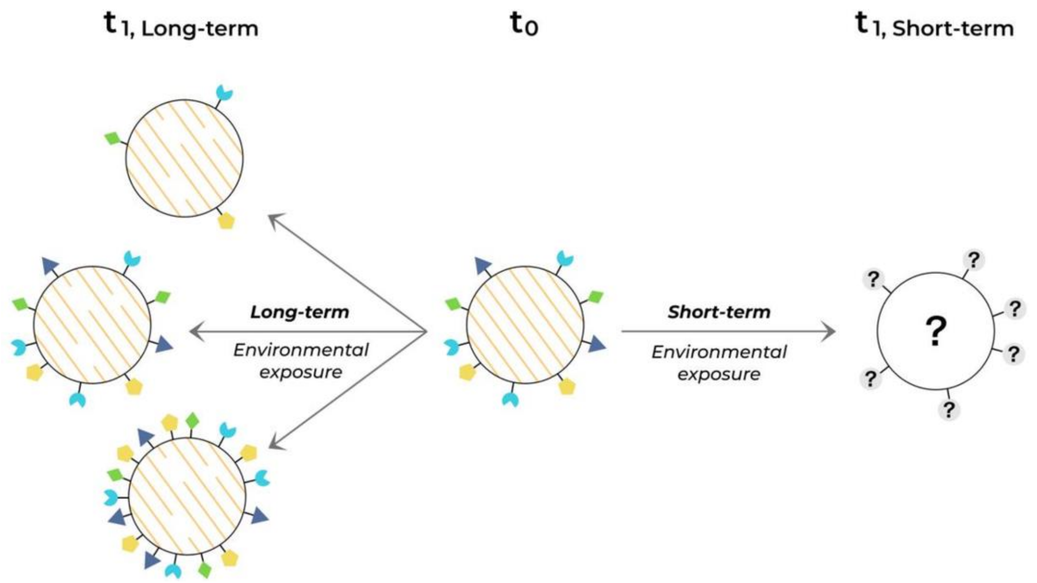

Figure S19. Environmental Exposure degrades polymer and changes surface reactivity as well as diffusion rates and uptake probabilities. Long-term degradation may lead to lower, equal or higher number of SFGs, while also degrading the bulk of the material. The potential outcome after short-term degradation are unknown to this point.
